# A Novel Signature of Disulfidptosis‐Related lncRNAs Predicts Prognosis in Glioma: Evidence From Bioinformatic Analysis and Experiments

**DOI:** 10.1155/ijog/5573323

**Published:** 2025-10-13

**Authors:** Taiyao Li, Ying Cao, Jie Wang, Xiaoyuan Tian, Bin Dong, Yanqin Yang, Pengpeng Zhang

**Affiliations:** ^1^ Department of Neurosurgery, The First Affiliated Hospital of Dalian Medical University, Dalian, Liaoning, China, dlmedu.edu.cn; ^2^ Department of Radiation Oncology, The Second Affiliated Hospital of Dalian Medical University, Dalian, Liaoning, China, dlmedu.edu.cn; ^3^ Department of Thoracic Tumor Radiotherapy, Shandong First Medical University Cancer Hospital, Jinan, Shandong, China

**Keywords:** disulfidptosis, glioma, LINC02542, prognostic signature, tumor immune microenvironment

## Abstract

Glioma is the most common primary malignant brain tumor, characterized by high mortality and poor prognosis. Disulfidptosis, a recently identified form of regulated cell death, has been implicated in tumor progression; however, its role in glioma remains unclear. In this study, we developed and validated a novel prognostic signature based on disulfidptosis‐related long noncoding RNAs (DRLs) by integrating transcriptomic and clinical data from The Cancer Genome Atlas. Seven DRLs were identified to construct a risk model that effectively stratified patients into high‐ and low‐risk groups with significantly different overall survival outcomes. Functional enrichment and immune‐related analyses revealed that the high‐risk group exhibited distinct immune microenvironment features, including altered immune cell infiltration, immune checkpoint expression, and activity of immune‐related pathways, suggesting a potential link between DRLs and immune modulation. Drug sensitivity analysis identified several chemotherapeutic agents and targeted inhibitors with higher predicted efficacy in the high‐risk group, offering insights into personalized treatment strategies. In vitro experiments further demonstrated that LINC02542 knockdown significantly suppressed glioma cell proliferation, migration, and invasion. Collectively, these findings indicate that the DRL signature functions as an independent prognostic indicator and a potential biomarker for immune landscape profiling and immunotherapy response prediction in glioma. This integrative multiomics approach provides novel perspectives for precision immunotherapy and targeted therapy in glioma.

## 1. Introduction

Gliomas are central nervous system tumors originating from glial cells and account for approximately 75% of all primary malignant brain tumors in adults, with around 80% classified as high grade and 20% as low grade [1]. High‐grade gliomas are markedly more aggressive and associated with poorer clinical outcomes than low‐grade gliomas (LGGs) [2]. The standard treatment for both primary and recurrent gliomas includes maximal safe surgical resection followed by radiotherapy combined with concurrent temozolomide, as recommended by the Stupp protocol [3]. Although this regimen improves survival, particularly in patients with isocitrate dehydrogenase (IDH) mutations, its efficacy is limited in most high‐grade gliomas [3]. Despite advances in molecularly targeted therapies for various malignancies, only a small subset of glioma patients benefit from these approaches [4]. Immunotherapy including immune checkpoint inhibitors, cytokine‐based treatments, and oncolytic viruses has shown promise in preclinical studies and clinical trials; however, further investigation is needed to determine its efficacy across molecular subtypes of glioma.

Regulated cell death (RCD) is a gene‐driven, orderly form of cell death critical for maintaining tissue homeostasis and defending against pathological insults [5]. Various RCD subtypes, such as apoptosis, autophagy‐dependent cell death, necroptosis, and ferroptosis, influence tumor progression and therapeutic response [6]. Disulfidptosis, a novel form of metabolism‐associated RCD, was first described in March 2023 [7]. It is mediated by SLC7A11, a cystine–glutamate antiporter. Under glucose‐starved conditions and high SLC7A11 expression, cystine accumulation driven by NADPH depletion results in abnormal disulfide bond formation within the cytoskeleton, causing F‐actin contraction and cytoskeletal collapse, ultimately leading to cell death. Notably, disulfidptosis is not inhibited by known cell death inhibitors and can only be reversed by disulfide bond‐disrupting agents such as *β*‐mercaptoethanol [7]. Emerging evidence suggests that dysregulation of disulfidptosis‐related genes (DRGs) may influence key cancer pathways, tumor microenvironment (TME) dynamics, and tumor stemness [8]. Given its role in tumor progression and immune modulation, disulfidptosis may represent a novel therapeutic target for improving the treatment outcomes of glioma.

Long noncoding RNAs (lncRNAs) are longer than 200 nucleotides that do not encode proteins. They are abundantly expressed in the central nervous system [9] and exhibit spatiotemporal regulation during development [10]. Aberrant lncRNA expression has been implicated in various neurological disorders [11]. Functionally, lncRNAs can modulate oncogenic processes such as proliferation, invasion, metastasis, inhibition of cell death, and alterations in DNA damage responses [12]. lncRNAs may act as oncogenes or tumor suppressors, for example, NEAT1 promoting glioma progression by stabilizing PGK1 [13]. lncRNA profiling also holds clinical importance for glioma subtyping and prognostication. Li et al. identified three molecular subtypes of glioma using lncRNA‐based molecular subclassification, each with astrocytomas with high EGFR amplification, neuronal tumors, and IDH‐mutant oligodendrogliomas with 1p19q codeletion, significantly correlating with patient survival [14]. These findings underscore the importance of lncRNA profiling in glioma biology and clinical management. While several DRGs have been characterized, to our knowledge, the prognostic significance of disulfidptosis‐related lncRNAs (DRLs) and their correlation with the tumor immune landscape in gliomas remain poorly understood. Given the extensive involvement of lncRNAs in central nervous system pathophysiology, it is necessary to investigate DRLs in gliomas and understand their potential clinical significance.

In this study, we employed a bioinformatics approach to construct and validate a prognostic model comprising seven DRLs for predicting clinical outcomes in glioma patients. We further assessed the impact of the DRL signature on tumor‐related pathways, immune microenvironment features, and drug sensitivity. Finally, we experimentally verified the role of LINC02542 in promoting glioma malignancy, establishing it as a promising prognostic biomarker and potential therapeutic target. A non–peer‐reviewed version of this manuscript was posted on Research Square [15]; the current submission presents the same scientific content but has undergone professional language editing and is submitted here for formal peer review.

## 2. Materials and Methods

### 2.1. Data Collection and Identification of DRLs

Transcriptomic, clinical, and single nucleotide polymorphism (SNP) data of glioma patients, including those with LGG and glioblastoma multiforme (GBM), were retrieved from The Cancer Genome Atlas (TCGA, https://www.tcga.org/), comprising 704 samples (last accessed: 24 July 2024). Based on previously published studies, 24 DRGs were identified (Table S1) [7, 16–19]. Then, Pearson correlation analysis was conducted to identify lncRNAs (|*r*| > 0.4, *p* < 0.001).

### 2.2. Construction of the DRL Prognostic Signature

The 704 glioma samples were randomly divided into training and test sets in a 1:1 ratio. The training set was used to construct the DRL prognostic model, while the test set and entire cohort were used for validation. Univariate Cox regression analysis was first employed to identify survival‐associated DRLs. These were further refined using the least absolute shrinkage and selection operator (LASSO) method with 10‐fold cross‐validation (*p* < 0.05), followed by multivariate Cox regression analysis to identify DRLs independently associated with overall survival (OS). These analyses were performed using the R packages “caret,” “glmnet,” [20] “survival,” and “survminer.” A prognostic risk score was then calculated for each sample using the formula:

Risk score=∑inExpi∗βi

where *E* *x* *p* *i* represents the expression level and *β* *i* the corresponding regression coefficient of each DRL. Based on the median risk score, patients were stratified into high‐ and low‐risk groups.

### 2.3. Prognostic Model Verification and Clinical Correlation Analysis

The prognostic performance of the DRL model was evaluated using Kaplan–Meier (K‐M) curves for OS and progression‐free survival (PFS) in the test set and the entire cohort. K‐M analysis was also conducted across clinical subgroups stratified by age, gender, and tumor grade. Model performance was further assessed using receiver operating characteristic (ROC) curves and the concordance index (*C*‐index).

### 2.4. Independent Prognostic Analysis, Nomogram Construction, and Principal Component Analysis (PCA)

Univariate and multivariate Cox regression analyses (*p* < 0.001) were performed to determine whether the risk score is an independent prognostic factor for OS. A nomogram predicting 1‐, 3‐, and 5‐year OS was constructed using the R package “RMS” [21], and its predictive accuracy was evaluated with calibration curves. PCA was conducted using the “scatterplot3d” R package to visualize expression profiles based on the whole transcriptome, DRGs, all DRLs, and model‐included DRLs [22].

### 2.5. Differential Gene Expression and Enrichment Analysis

Differentially expressed genes (DEGs) between high‐ and low‐risk groups were identified using the R package “limma” (|log_2_ fold change (FC) | > 1, adjusted *p* < 0.05). Subsequently, enrichment analysis and gene set enrichment analysis (GSEA) based on the Kyoto Encyclopedia of Genes and Genomes (KEGG) [23] and Gene Ontology (GO) [24] were conducted to identify pathways and functions enriched by DEGs. Gene set variation analysis (GSVA) was also performed using KEGG and Hallmark reference gene sets to identify pathways associated with cancer phenotypes. The analyses utilized the “clusterProfiler,” “org.Hs.eg.db,” and “GSVA” R packages.

### 2.6. Immune Landscape Analysis

Immune infiltration analyses were conducted using the R package “IOBR,” which integrates seven algorithms (Xcell, Timer, Quantiseq, MCPcounter, EPIC, CIBERSORT‐ABS, and CIBERSORT). These were used to explore the correlation between immune cell infiltration and risk scores and to compare immune cell infiltration patterns between the two risk groups. Single‐sample GSEA (ssGSEA) and GSVA were used to assess differences in immune‐related pathways and functions. The expression levels of immune checkpoint‐related genes were compared using *t*‐tests. Stromal, immune, and ESTIMATE scores were calculated using the “estimate” R package to evaluate differences in the TME composition [25]. The Tumor Immunity Dysfunction and Exclusion (TIDE) platform (http://tide.dfci.harvard.edu/) was used to assess tumor immune evasion and immunotherapy responsiveness [26]. Immune checkpoint gene expression levels were also analyzed across the risk groups.

### 2.7. TMB Analysis and Drug Sensitivity Assessment

The R package “maftools” was used to assess tumor mutational burden (TMB) differences between high‐ and low‐risk groups, and Spearman correlation was applied to analyze associations between TMB and risk scores. K‐M analysis was used to assess the combined effect of TMB and risk scores on OS. Drug sensitivity predictions based on half‐maximal inhibitory concentration (IC_50_) were performed using the R package “oncoPredict” [27], and group differences in response to chemotherapy and targeted agents were compared using Wilcoxon tests.

### 2.8. Consensus Clustering Analysis

To explore DRL‐based molecular subtypes with potential immunotherapy relevance, consensus clustering was performed using expression data from the DRL model. The optimal number of clusters (*k*) was determined using the cumulative distribution function (CDF). Clustering was performed with the R package “ConsensusClusterPlus” [28]. OS, TME composition, and immune function characteristics were compared across clusters.

### 2.9. DRL Expression Differential Analysis and Subcellular Localization Prediction

The expression levels of DRLs in glioma versus normal brain tissue were analyzed using GEPIA2 (http://gepia2.cancer-pku.cn/) [29], which integrates data from 518 LGG, 163 GBM, and 207 normal brain tissue samples from TCGA and GTEx databases. Subcellular localization of LINC02542 was predicted using lncLocator (http://www.csbio.sjtu.edu.cn/bioinf/lncLocator/) [30].

### 2.10. Cell Lines and Cell Culture

U251 and U87 were obtained from the Cell Resource Center, Peking Union Medical College (PCRC, China). Cells were cultured in high‐glucose DMEM (Gibco, United States) supplemented with 10% fetal bovine serum and 1% penicillin–streptomycin (Beyotime, China).

### 2.11. Cell Transfection and Reverse Transcription–Quantitative PCR (RT‐qPCR)

Four siRNAs targeting LINC02542 (si‐LINC02542) and a negative control (si‐NC) were synthesized by GenePharma (Shanghai, China). Transfections were performed using jetPRIME (Polyplus, France) following the manufacturer’s protocol. Briefly, 300,000 cells were seeded per well in six‐well plates. A mixture containing siRNA (50 nM final concentration), 200 *μ*L jetPRIME buffer, and 4 *μ*L jetPRIME reagent was vortexed and incubated at room temperature for 10 min before being added to the culture medium. After 24 h of incubation at 37°C and 5% CO_2_, knockdown efficiency was assessed by RT‐qPCR. Total RNA was extracted using Trizol, and gene expression was normalized to hydroxymethylbilane synthase (HMBS) mRNA using the 2^−*ΔΔ*CT^ method [31]. siRNA sequences and primer details are provided in Supporting Information 1: Tables S2 and S3, respectively.

### 2.12. Cell Counting Kit‐8

Cell proliferation was assessed using the CCK‐8 assay. Transfected and control cells were seeded into 96‐well plates at 1500 cells/well. On Days 0, 1, 2, and 3, 10 *μ*L CCK‐8 reagent (Beyotime, China) was added to each well, and absorbance was measured at 450 nm after 2 h of incubation. All experiments were performed in triplicate.

### 2.13. Transwell Assay

Cell migration and invasion were assessed using Transwell chambers. For migration, 10,000 transfected or control cells in 100 *μ*L of serum‐free medium were seeded in the upper chamber of a 24‐well plate, with 700 *μ*L of 10% FBS medium in the lower chamber. For invasion assays, chambers were precoated with Matrigel (1:6 dilution). After 48 h of incubation at 37°C, cells on the lower surface were fixed with 4% paraformaldehyde, stained with crystal violet, and counted in five randomly selected fields under an inverted microscope (Nikon).

### 2.14. Statistical Analysis

Pearson correlation coefficients were used for correlation analyses. Group comparisons were performed using the chi‐square test or independent samples *t*‐test, as appropriate.

## 3. Results

### 3.1. Identification and Establishment of a Prognostic Signature for DRLs

**Figure 1:**
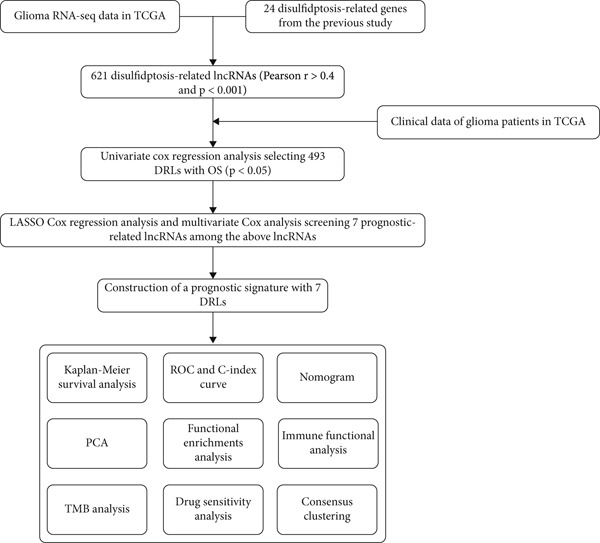
Workflow of the study.

The study workflow is illustrated in Figure 1. A total of 16,876 lncRNAs were obtained from 704 glioma samples in the TCGA database. Using Pearson correlation analysis (*r* > 0.4, *p* < 0.001), 621 DRLs were identified as being significantly associated with the expression of 24 DRGs. The expression correlations between DRGs and DRLs are visualized in a Sankey diagram (Supporting Information 2: Figure S1a).

Univariate Cox regression identified 493 DRLs significantly associated with OS (*p* < 0.001). To minimize overfitting, LASSO regression with 10‐fold cross‐validation was applied, yielding 18 prognostically relevant DRLs (*p* < 0.05) (Supporting Information 2: Figure S1b–d). Subsequent multivariate Cox regression reduced the model to seven DRLs: AC002456.1, AC010273.3, AC010884.1, AL138479.2, DNAJC3‐DT, FAM53B‐AS1, and LINC02542 (*p* < 0.05). A heatmap showed expression correlations between these seven DRLs and the 24 DRGs (Supporting Information 2: Figure S1e), based on which a prognostic model was constructed.

### 3.2. Validation of the DRL Signature and Correlation With Clinical Features

Glioma patients were stratified into high‐ and low‐risk groups using the median risk score from the training cohort. K‐M analysis of the training, test, and full datasets revealed significantly longer OS and PFS in the low‐risk group (Figures 2a, 2b, and 2c; Supporting Information 3: Figure S2a–c). Mortality increased with rising risk scores (Figures 2d, 2e, 2f, 2g, 2h, and 2i). Heatmaps indicated that four DRLs (AC002456.1, LINC02542, FAM53B‐AS1, and AC010273.3) were upregulated, while three (AC010884.1, DNAJC3‐DT, and AL138479.2) were downregulated in the high‐risk group (Figures 2j, 2k, and 2l).

Figure 2Validation of the DRL signature and correlation with clinical features. (a–c) Kaplan–Meier analysis of the training, test, and full datasets on OS. (d–f) Distribution of risk scores derived from the DRL model. (g–i) Survival time and survival status ranked by risk score. (j–l) Heatmaps showing expression levels of seven DRLs in each patient.

**Figure:**
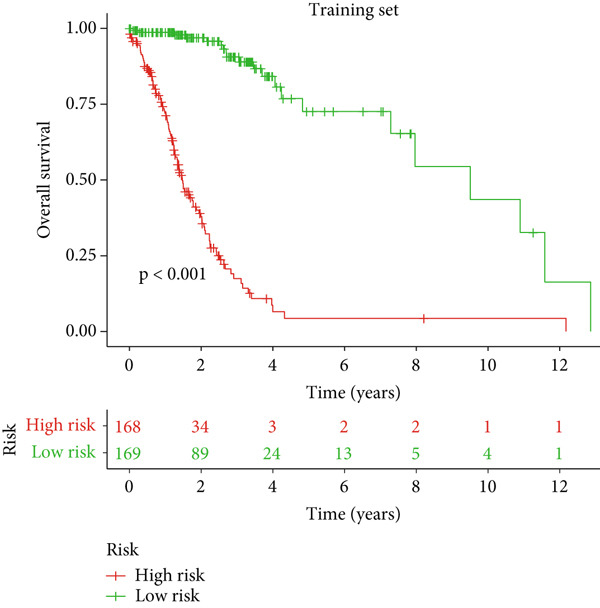
(a)

**Figure:**
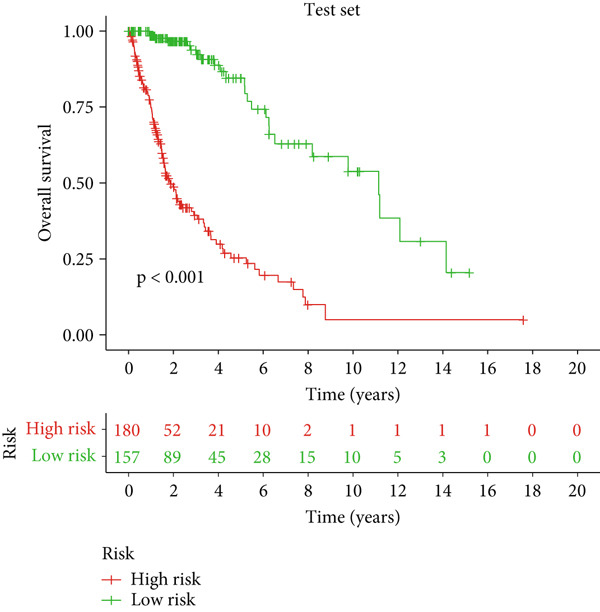
(b)

**Figure:**
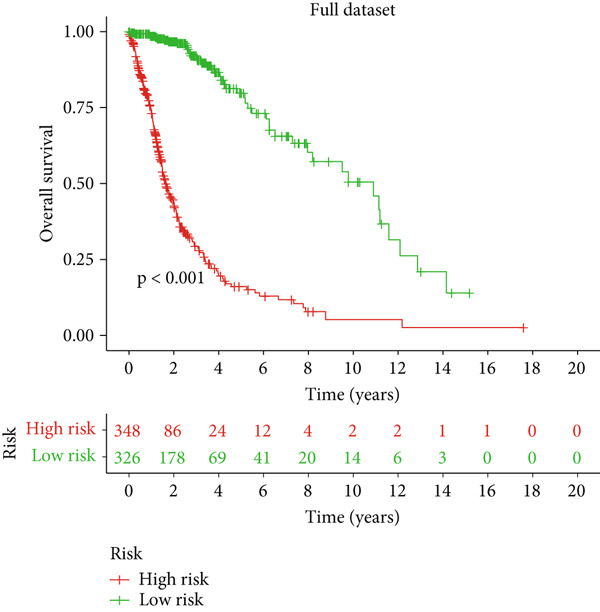
(c)

**Figure:**
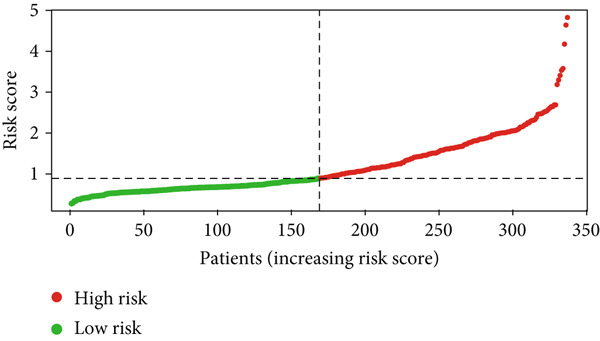
(d)

**Figure:**
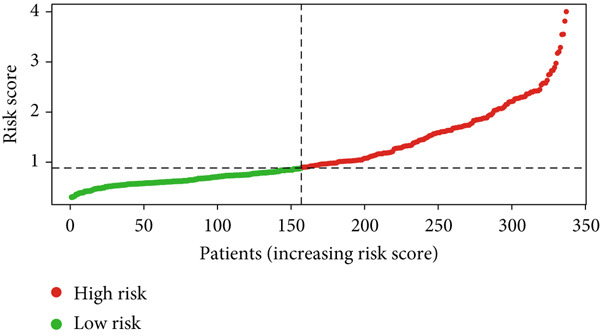
(e)

**Figure:**
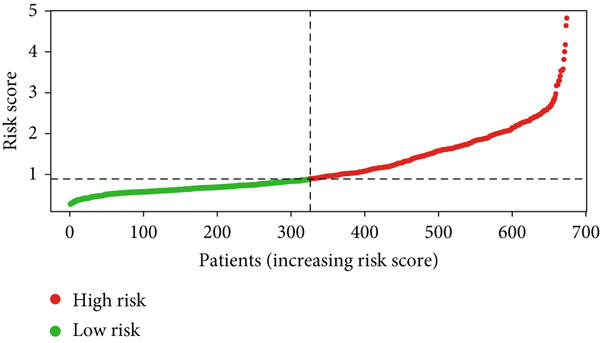
(f)

**Figure:**
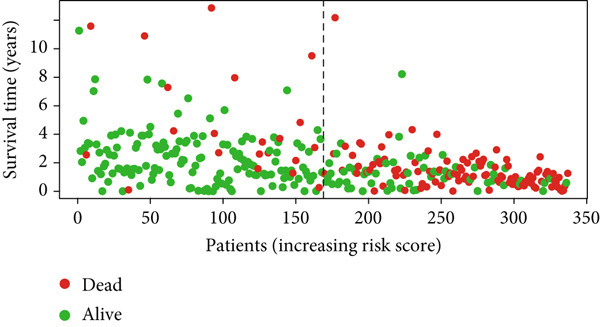
(g)

**Figure:**
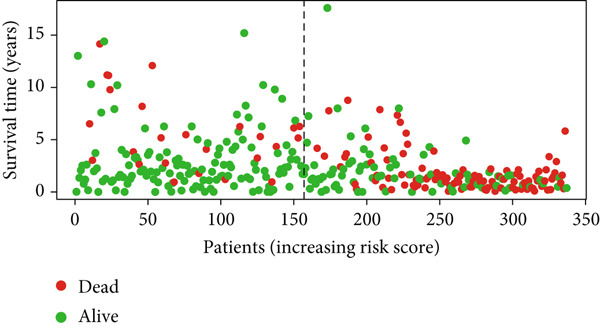
(h)

**Figure:**
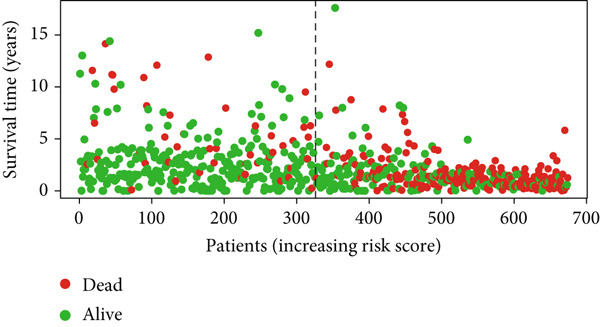
(i)

**Figure:**
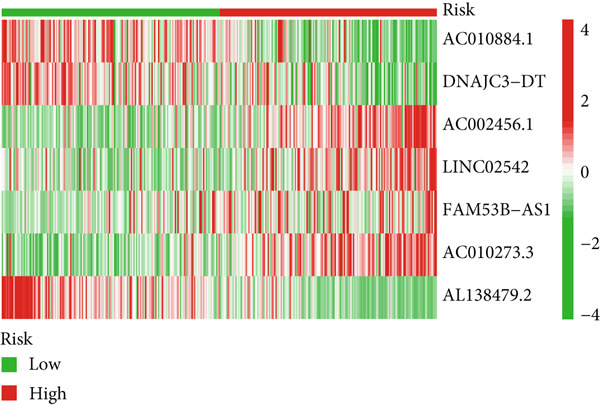
(j)

**Figure:**
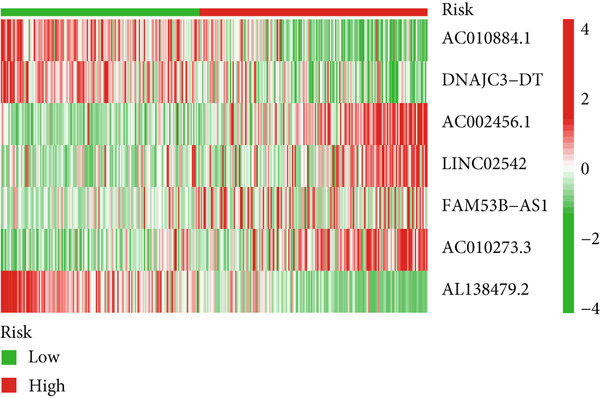
(k)

**Figure:**
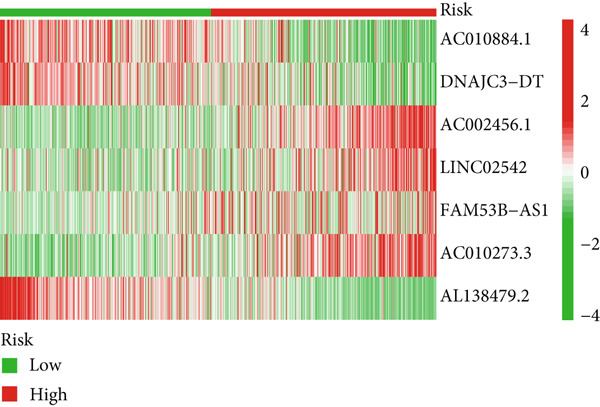
(l)

Clinical subgroup analyses confirmed that the high‐risk group contained a higher proportion of patients aged > 65 years and those with higher tumor grades (Supporting Information 4: Figure S3). K‐M analysis stratified by age, sex, and tumor grade consistently demonstrated superior OS in the low‐risk group (Figure 3 and Supporting Information 3: Figure S2d). ROC curves showed the DRL model had an area under the curve (AUC) of 0.914, outperforming age (0.824), gender (0.528), and tumor grade (0.765) (Figure 4c). The model also accurately predicted 1‐, 3‐, and 5‐year survival, with AUCs of 0.867, 0.914, and 0.888, respectively (Figure 4d). The *C*‐index of the model surpassed that of individual clinical features (Figure 4e), supporting its robustness as a prognostic tool.

Figure 3Correlation of the prognostic DRL signature with (a, b) age, (c, d) sex, and (e, f) tumor grade.

**Figure:**
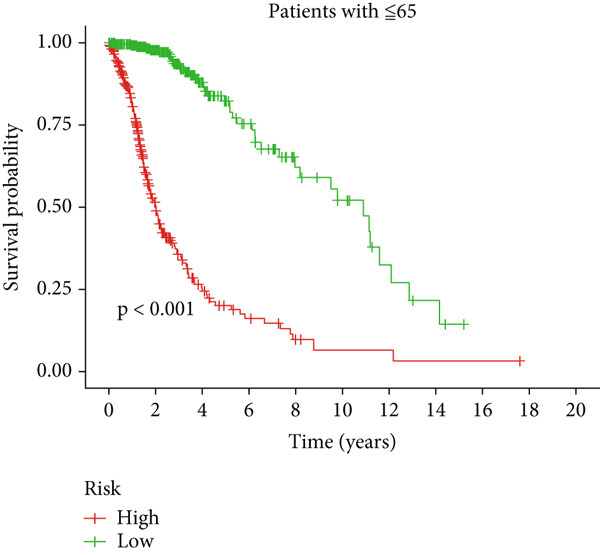
(a)

**Figure:**
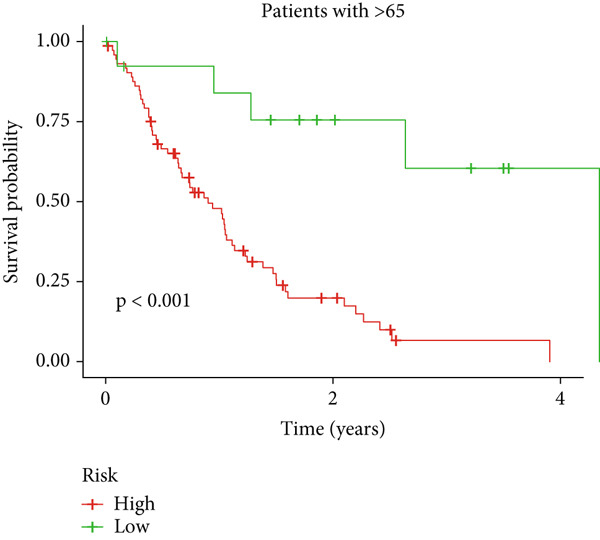
(b)

**Figure:**
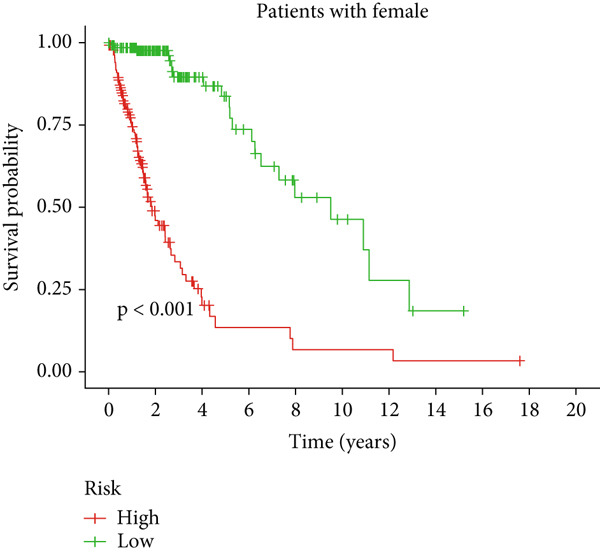
(c)

**Figure:**
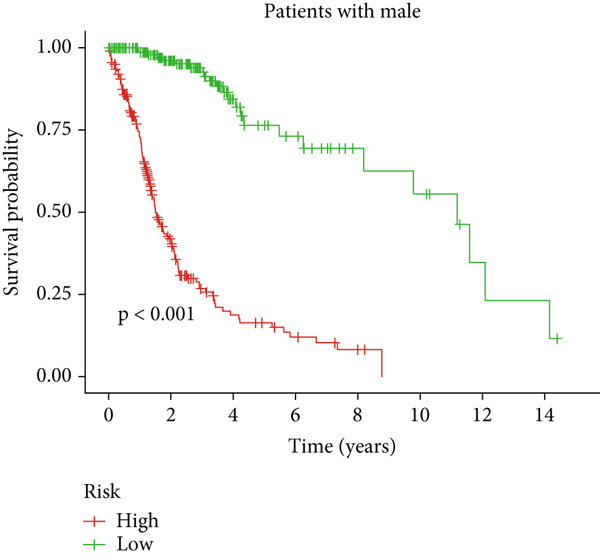
(d)

**Figure:**
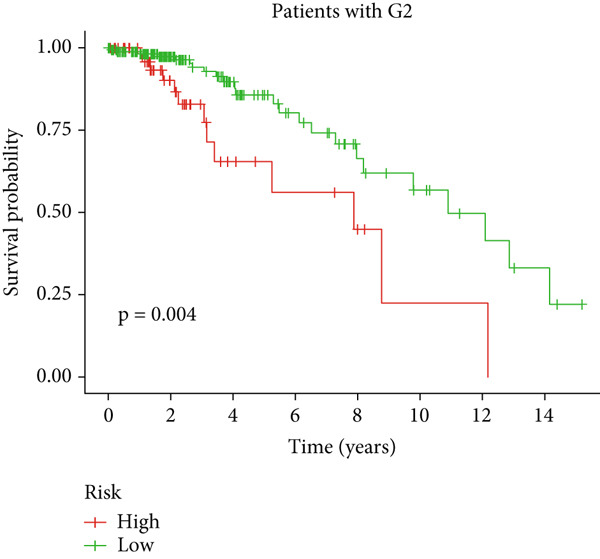
(e)

**Figure:**
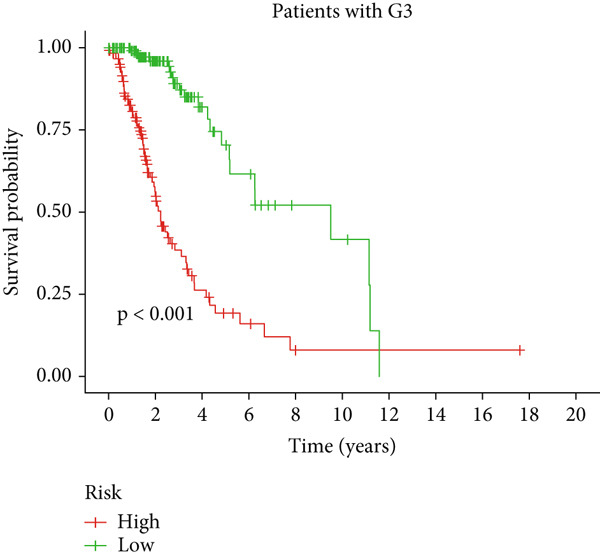
(f)

Figure 4DRL model performance evaluation and nomogram construction. (a) Univariate Cox regression analysis. (b) Multivariate Cox regression analysis. (c) ROC curves. (d) AUC curves for 1‐, 3‐, and 5‐year survival rates. (e) Concordance index curves. (f) A nomogram predicting 1‐, 3‐, and 5‐year survival. (g) Calibration curves of the nomogram.  ^∗∗∗^ *p* < 0.001.

**Figure:**
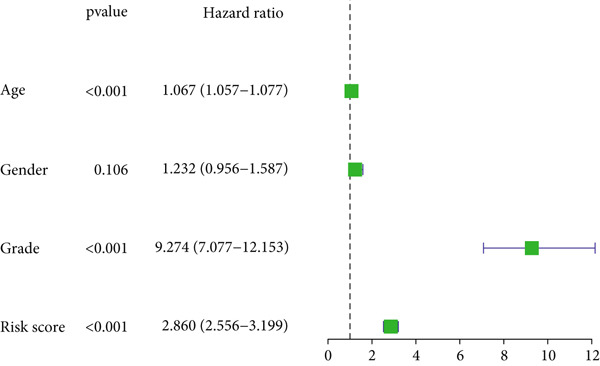
(a)

**Figure:**
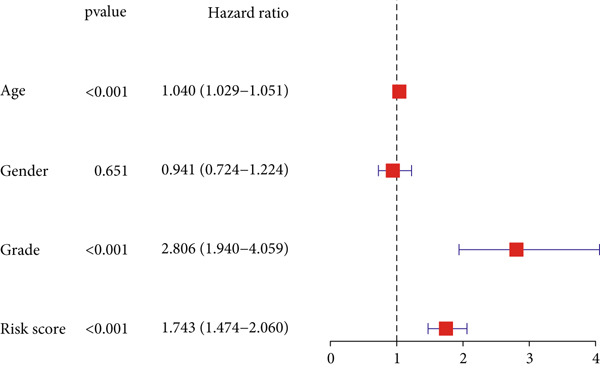
(b)

**Figure:**
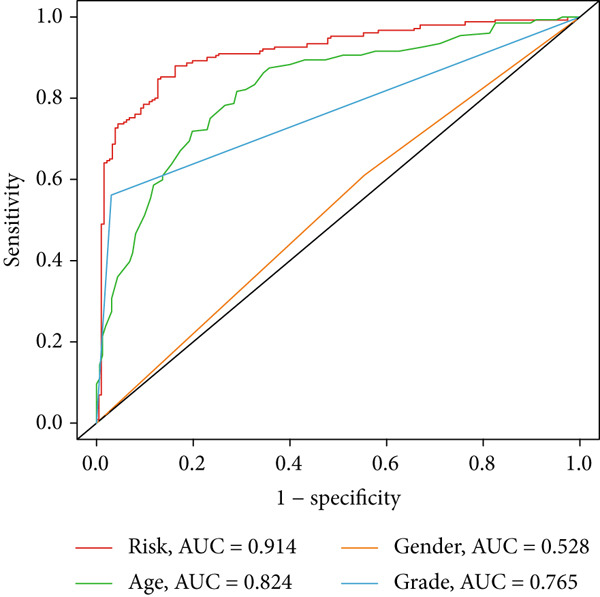
(c)

**Figure:**
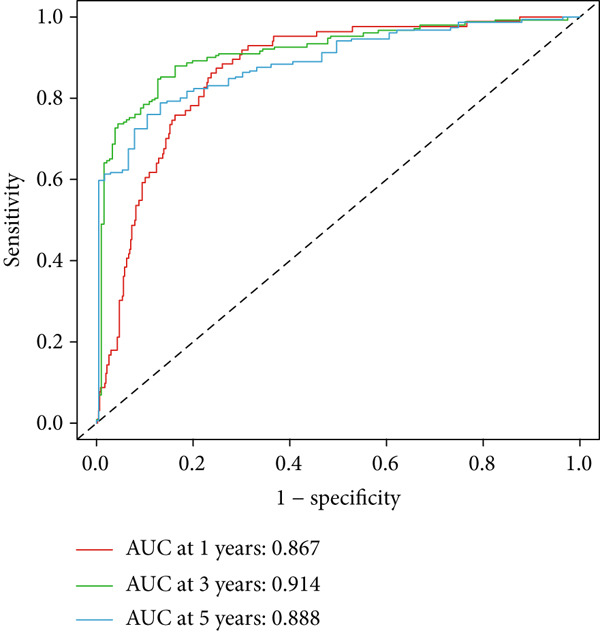
(d)

**Figure:**
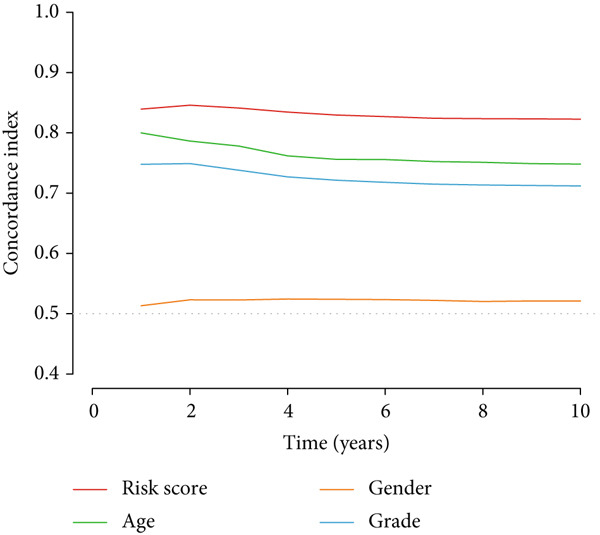
(e)

**Figure:**
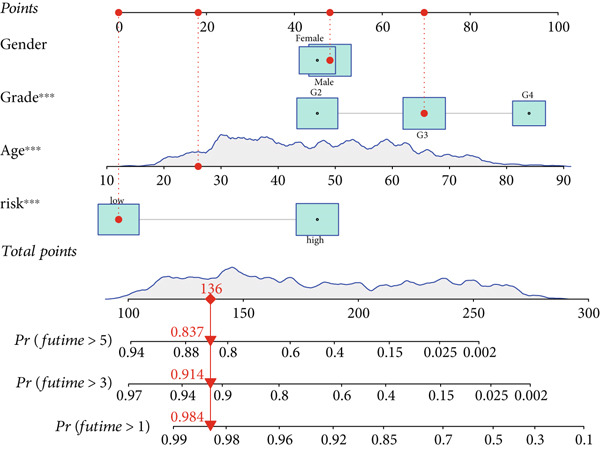
(f)

**Figure:**
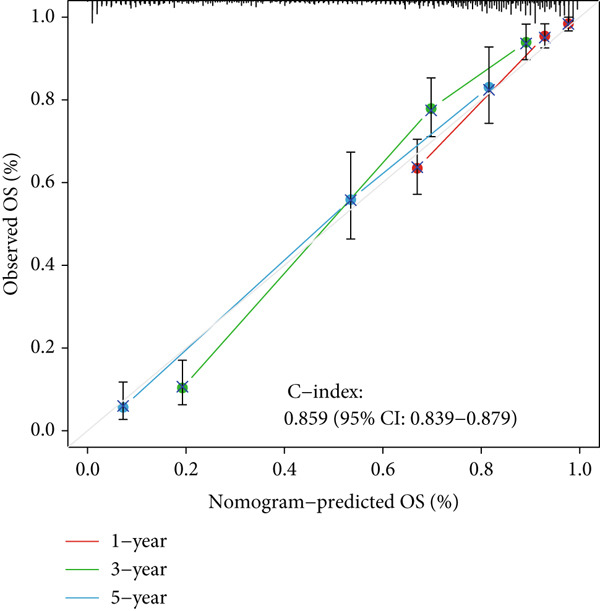
(g)

### 3.3. Construction of a Nomogram and PCA

Univariate and multivariate Cox regression analyses were performed to assess whether the DRL signature independently predicted OS. Univariate analysis showed the risk score significantly predicted OS (HR = 2.860, 95% CI: 2.556–3.199, *p* < 0.001; Figure 4a). Multivariate analysis confirmed the risk score as an independent prognostic factor after adjusting for age, gender, and tumor grade (HR = 1.743, 95% CI: 1.474–2.060, *p* < 0.001; Figure 4b). A nomogram integrating age, gender, tumor grade, and risk score was constructed to estimate 1‐, 3‐, and 5‐year OS (Figure 4f). For a total score of 136, the estimated survival rates were 0.984, 0.914, and 0.837, respectively. Calibration curves showed good agreement between predicted and observed survival, with a *C*‐index of 0.859 (95% CI: 0.839–0.879) (Figure 4g). PCA based on expression matrices of all genes, DRGs, DRLs, and the final DRL model demonstrated that the 7‐DRL signature achieved superior separation of high‐ and low‐risk groups (Supporting Information 5: Figure S4).

### 3.4. Differential Gene Expression and Functional Enrichment Analysis

Differential gene expression analysis identified 3498 genes significantly altered between risk groups (|log2 FC| > 1, FDR < 0.05), with 2710 genes upregulated and 788 downregulated in the high‐risk group (Figure 5a,b). KEGG pathway analysis revealed enrichment in neuroactive ligand–receptor interaction, cytokine–cytokine receptor interaction, phagosome formation, neutrophil extracellular trap formation, and antigen presentation (Figure 5c). GO analysis showed significant enrichment in functions such as antigen binding, immunoreceptor activity, MHC complex binding, and leukocyte‐mediated immunity (Figure 5d and Supporting Information 6: Figure S5a). GSEA further indicated enrichment in macrophage recognition, immunoglobulin complex formation, antigen binding, and receptor binding in the high‐risk group (Supporting Information 6: Figure S5b–e). The expression of the seven DRLs also correlated significantly with tumor‐associated pathways, including WNT, P53, and MAPK (Supporting Information 6: Figure S5f,g), suggesting a role in tumor progression and immune modulation.

Figure 5Differential gene expression and functional enrichment analysis. (a) Volcano plot showing gene expression differences between high‐ and low‐risk groups. (b) Heatmap of differentially expressed genes. (c) KEGG pathway enrichment analysis. (d) GO functional enrichment analysis.

**Figure:**
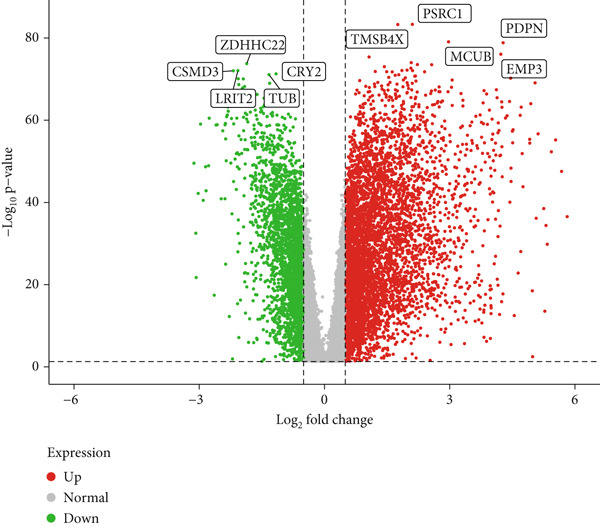
(a)

**Figure:**
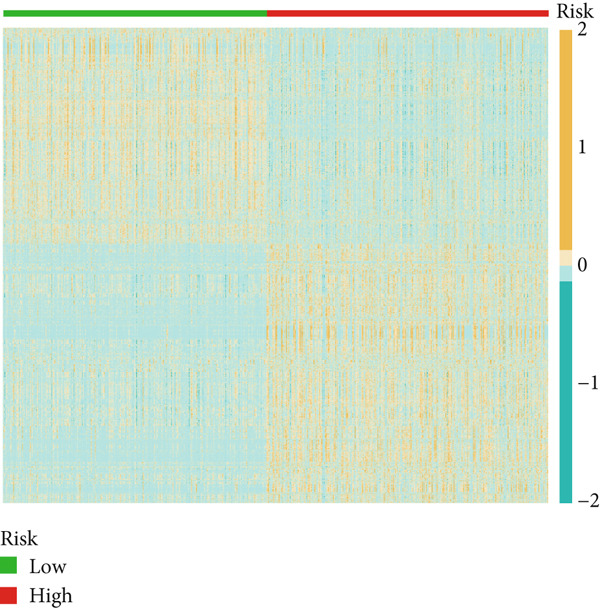
(b)

**Figure:**
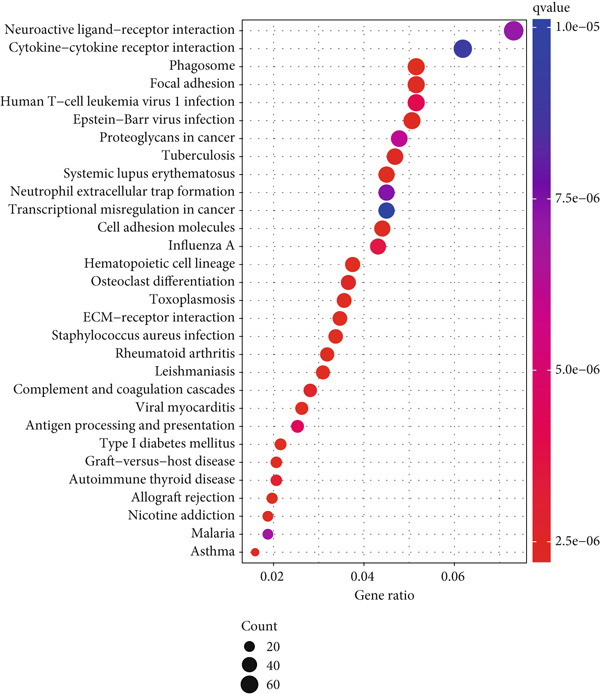
(c)

**Figure:**
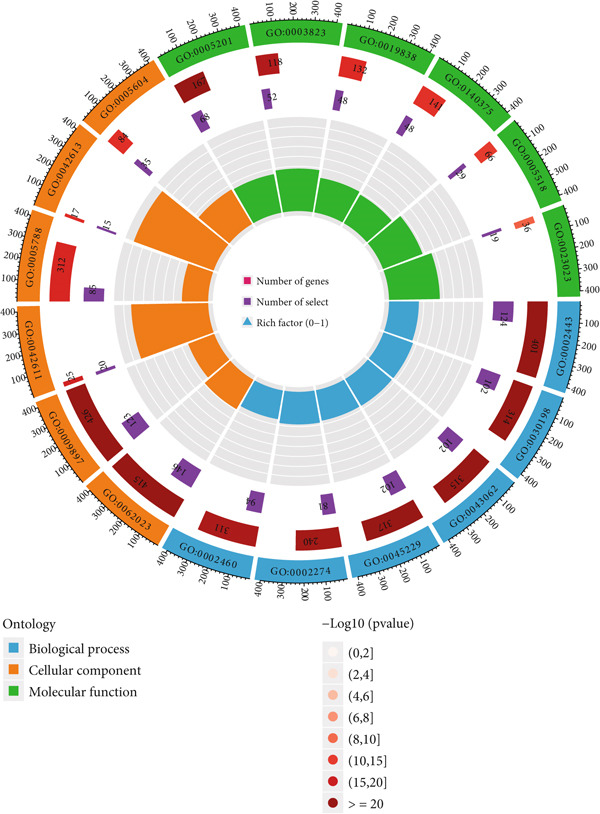
(d)

### 3.5. Immune‐Related Analysis in Different Risk Groups

Given the immune‐related enrichment of DEGs, immune profiling was performed. TME analysis revealed that stromal, immune, and ESTIMATE scores were significantly higher in the high‐risk group, indicating greater nontumor cell infiltration (Figure 6b). Seven immune infiltration algorithms demonstrated significant correlations between DRL risk scores and immune cell infiltration levels, particularly for macrophages, CD4^+^ T cells, and NK cells (Figure 6a and Supporting Information 7: Figure S6a). ssGSEA and GSVA analyses showed marked differences in immune pathway activities, including immune checkpoints and antigen‐presenting cell (APC) costimulation pathways between the two groups (Figure 6d and Supporting Information 7: Figure S6c). Subsequently, we used TIDE scores to study the tumor immune evasion ability of patients with glioma, which revealed significantly better abilities in the high‐risk group than in the low‐risk group (Figure 6c). Multiple immune checkpoint genes, including indoleamine 2,3‐dioxygenase 1 (IDO1), cytotoxic T lymphocyte‐associated protein 4 (CTLA4), and programmed cell death 1 ligand 1 (PD‐L1) (CD274), were expressed at higher levels in the high‐risk group (Figure 6e). The correlation analysis between DRLs and PD‐L1 showed varying degrees of expression differences in PD‐L1 among samples with high‐ and low DRL expression (*p* < 0.05) (Supporting Information 8: Figure S7). These findings highlight substantial immune microenvironment differences between risk groups and suggest potential for DRL‐guided immunotherapeutic strategies.

Figure 6Immune‐related analysis between high‐ and low‐risk groups. (a) Bubble chart showing correlations between risk score and immune cell infiltration based on seven algorithms. (b) Analysis of tumor microenvironment differences. (c) TIDE scores comparing tumor immune evasion. (d) ssGSEA analysis of immune function n pathways. (e) Differential expression analysis of immune checkpoint genes.  ^∗^ *p* < 0.05,  ^∗∗^ *p* < 0.01, and  ^∗∗∗^ *p* < 0.001.

**Figure:**
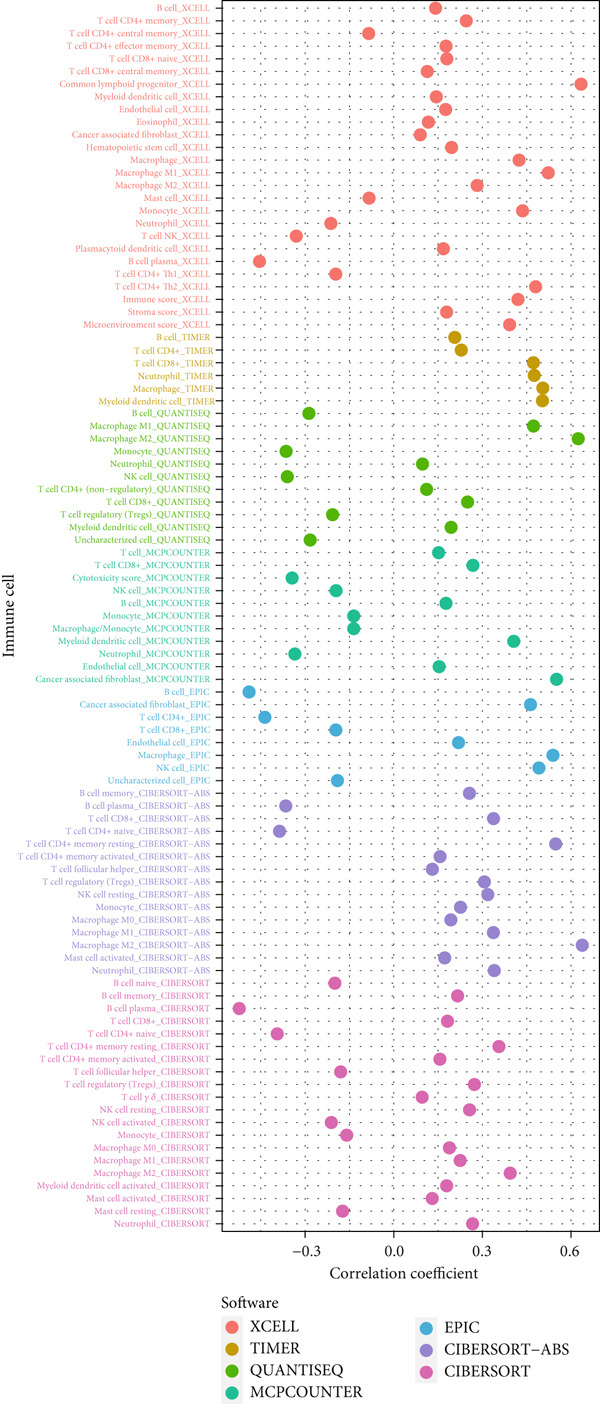
(a)

**Figure:**
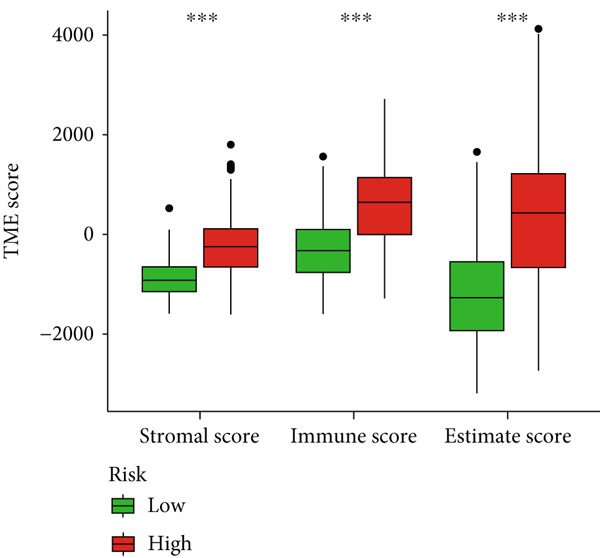
(b)

**Figure:**
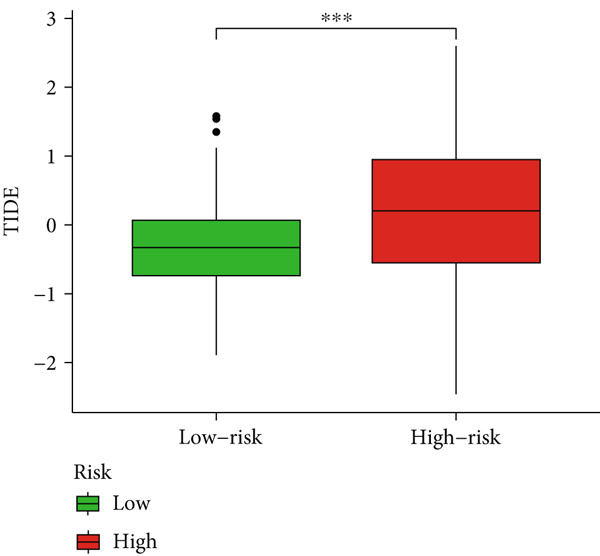
(c)

**Figure:**
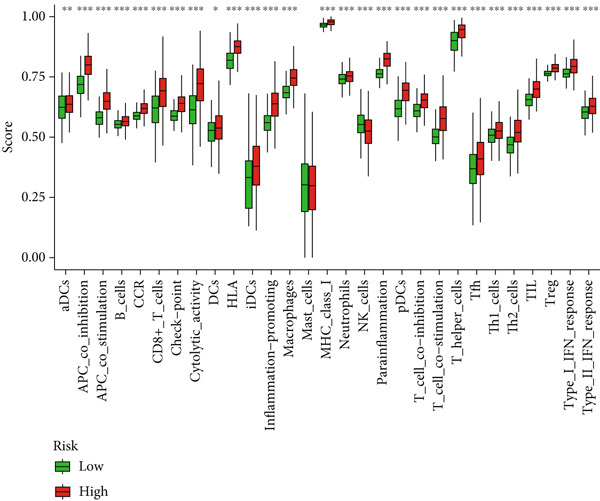
(d)

**Figure:**
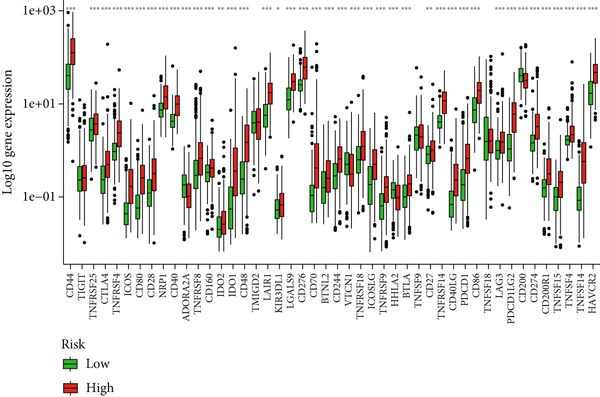
(e)

### 3.6. TMB Analysis and Drug Sensitivity Assessment

TMB was significantly higher in the high‐risk group and positively correlated with the risk score (Pearson *r* = 0.46, *p* < 0.001; Figure 7c,d). Mutation profiles indicated more frequent alterations in *PTEN*, *EGFR*, and *TTN* in the high‐risk group, whereas *IDH1* and *ATRX* mutations were more prevalent in the low‐risk group (Figure 7a,b). Survival analysis showed better OS in patients with low TMB (Figure 7e), with the best outcomes observed in those with both low TMB and low‐risk scores (Figure 7f).

Figure 7TMB analysis. (a, b) Top 15 most frequently mutated genes in high‐ and low‐risk groups. (c) TMB analysis between high‐ and low‐risk groups ( ^∗∗∗^ *p* < 0.001). (d) Correlation between TMB and risk scores. (e) Kaplan–Meier curves for overall survival in high‐ and low‐TMB groups. (f) Combined survival analysis of TMB and risk groups based on overall survival.

**Figure:**
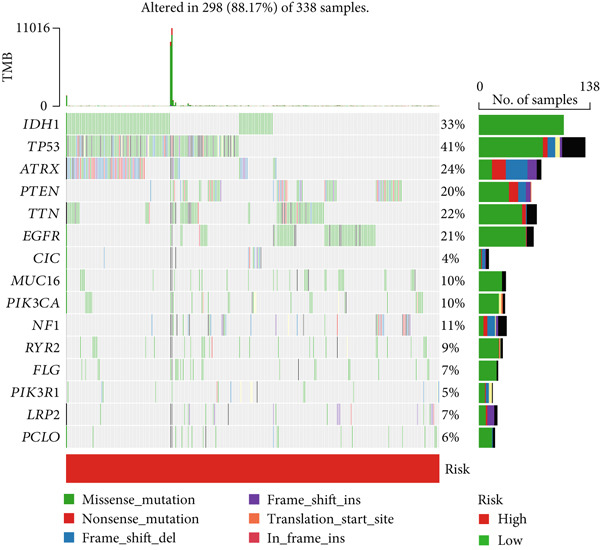
(a)

**Figure:**
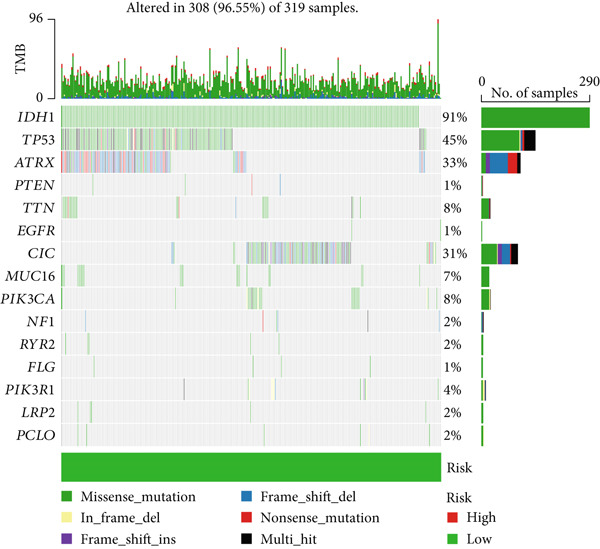
(b)

**Figure:**
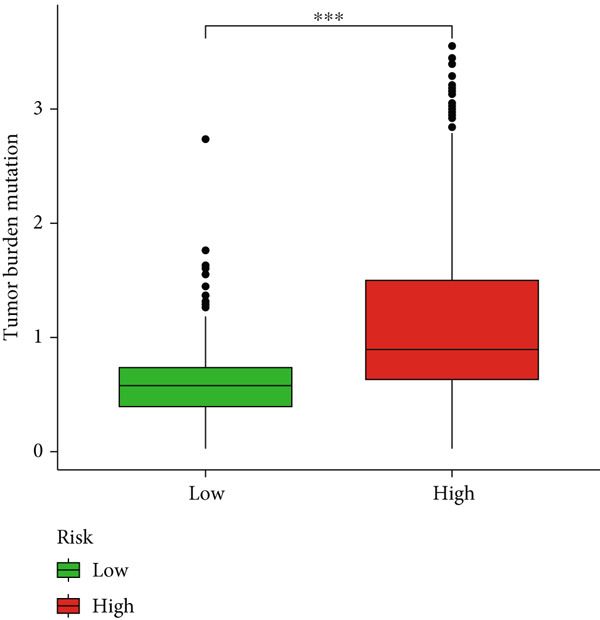
(c)

**Figure:**
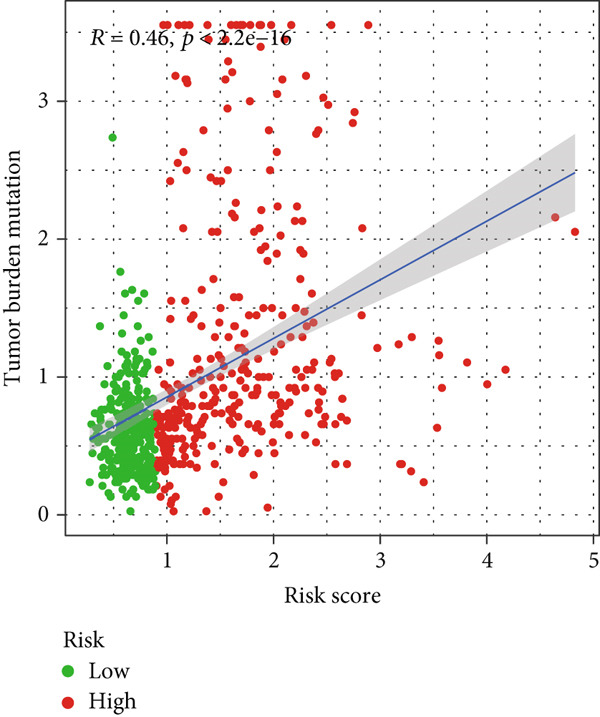
(d)

**Figure:**
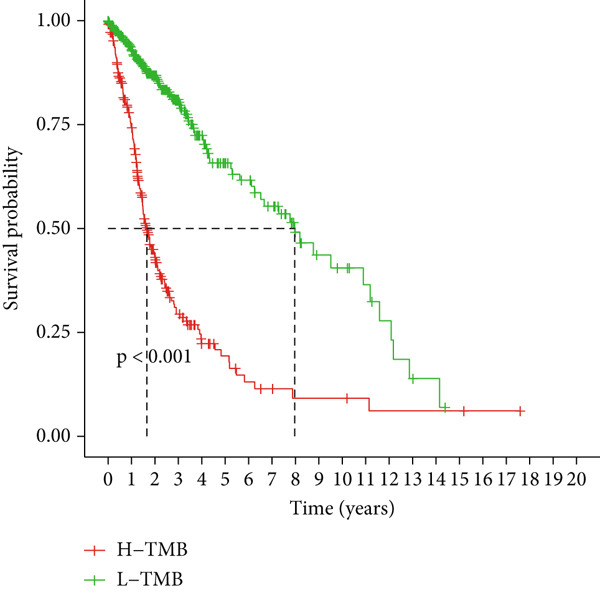
(e)

**Figure:**
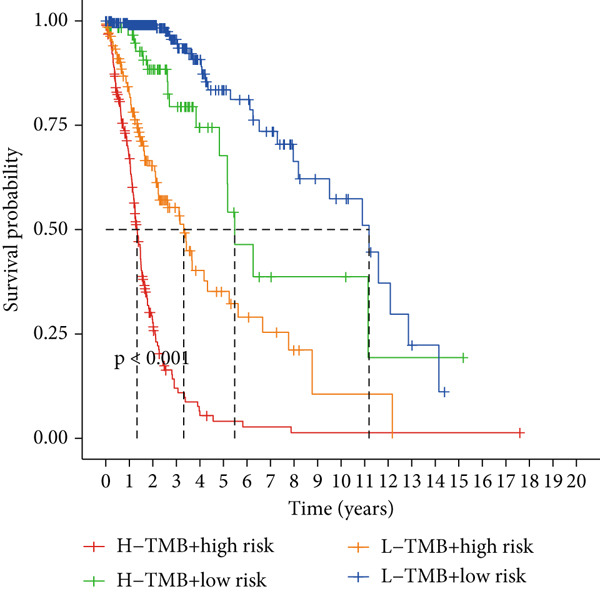
(f)

Drug sensitivity analysis revealed that the high‐risk group was more responsive to several agents, including 5‐fluorouracil, cisplatin, gemcitabine, and inhibitors targeting MEK/ERK, PI3K/AKT/mTOR pathways, BET proteins, DNA repair mechanisms, and HGFR. These agents demonstrated significantly lower IC_50_ values in the high‐risk group, indicating enhanced sensitivity (Supporting Information 9: Figure S8).

### 3.7. Consensus Cluster Analysis

To further characterize the immune landscape across glioma subtypes, consensus clustering based on DRL expression was performed, classifying 701 glioma samples into two clusters (Figure 8a,d). Heatmap analysis revealed distinct gene expression patterns between clusters (Figure 8b).

Figure 8Consensus clustering and immune‐related analyses. (a) Consensus clustering matrix (*k* = 2). (b) Heatmap showing differential gene expression between two clusters. (c) Sankey diagram illustrating associations among clusters, risk groups, and survival status. (d) Cumulative distribution function (CDF) curves. (e) Kaplan–Meier analysis for overall survival in the two clusters. (f) Tumor microenvironment differences between clusters. (g) ssGSEA showing differences in immune function pathways. (h) Differential expression of immune checkpoint genes.  ^∗^ *p* < 0.05,  ^∗∗^ *p* < 0.01, and  ^∗∗∗^ *p* < 0.001.

**Figure:**
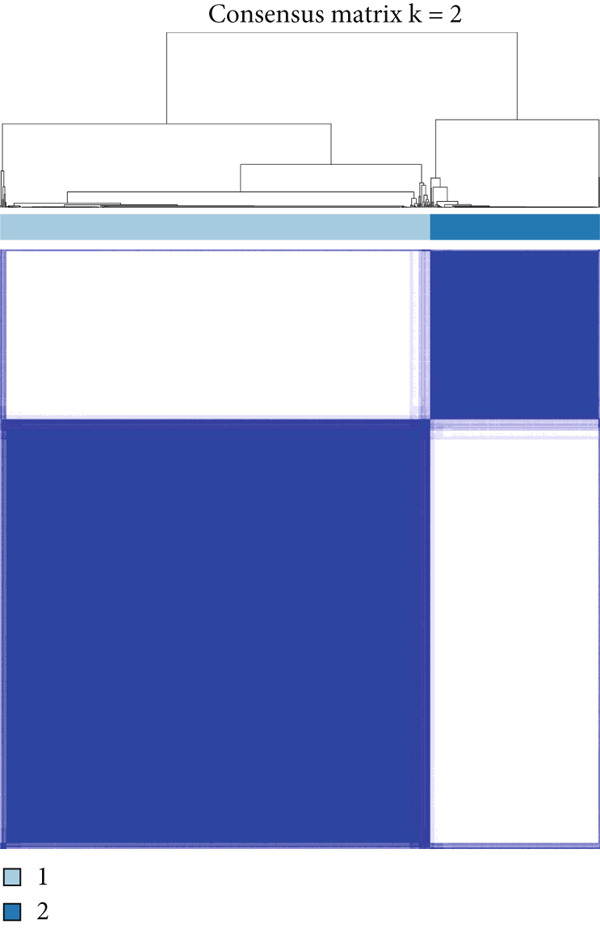
(a)

**Figure:**
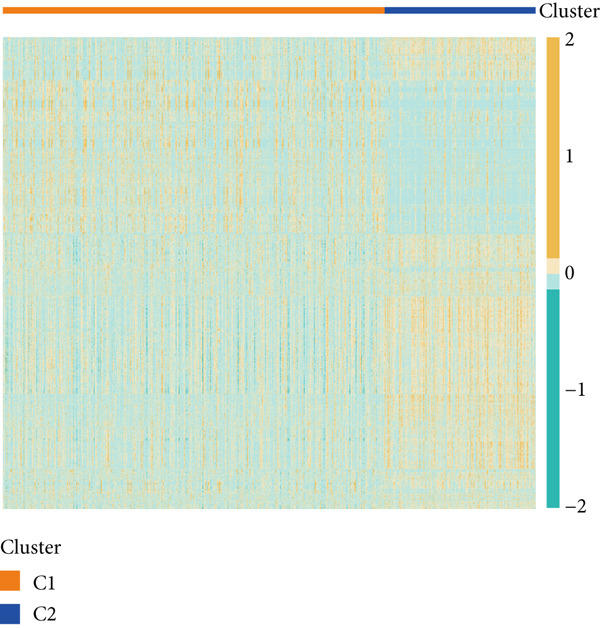
(b)

**Figure:**
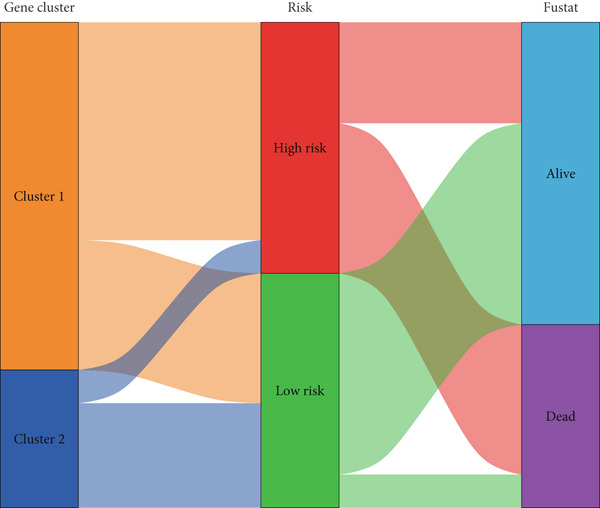
(c)

**Figure:**
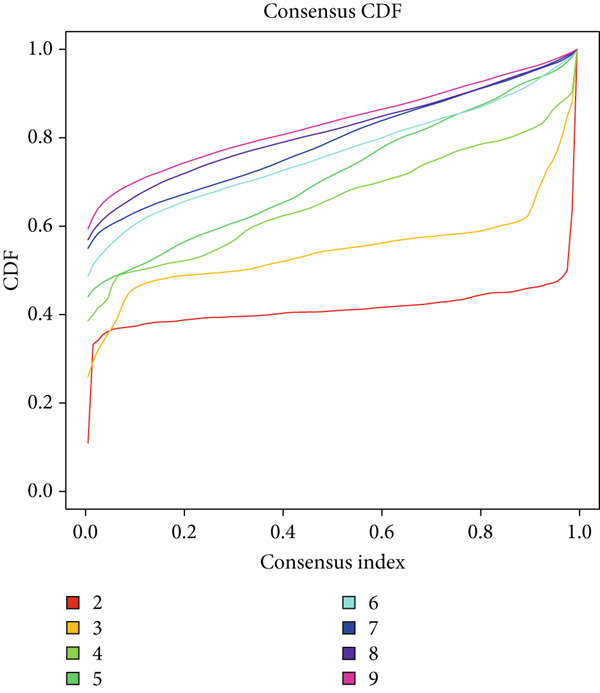
(d)

**Figure:**
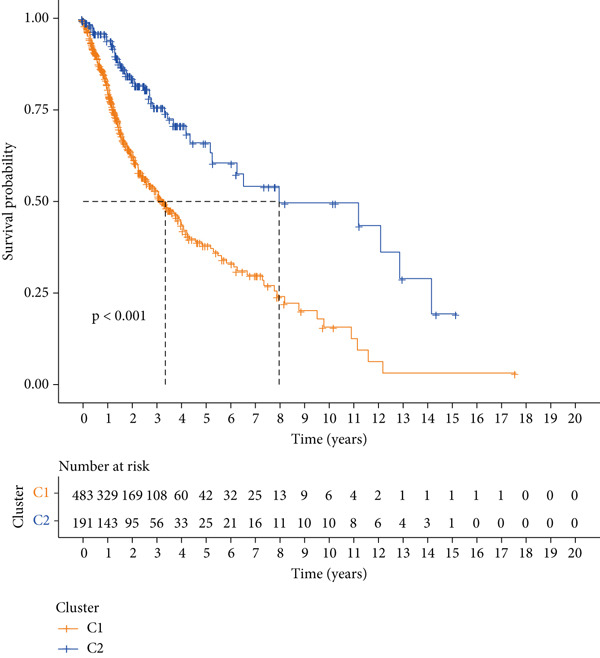
(e)

**Figure:**
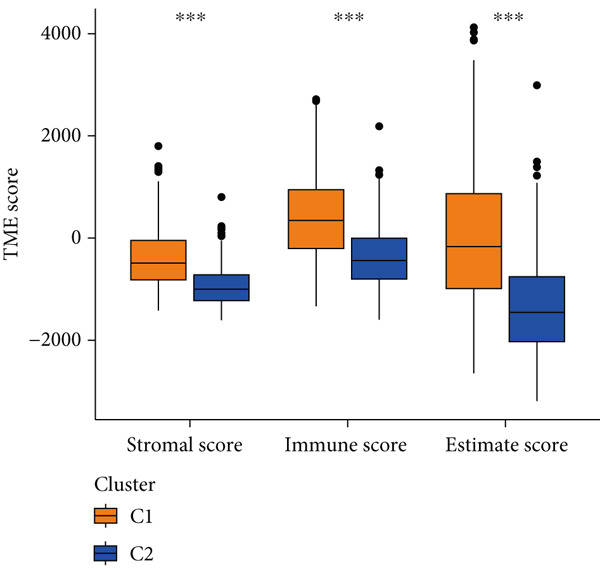
(f)

**Figure:**
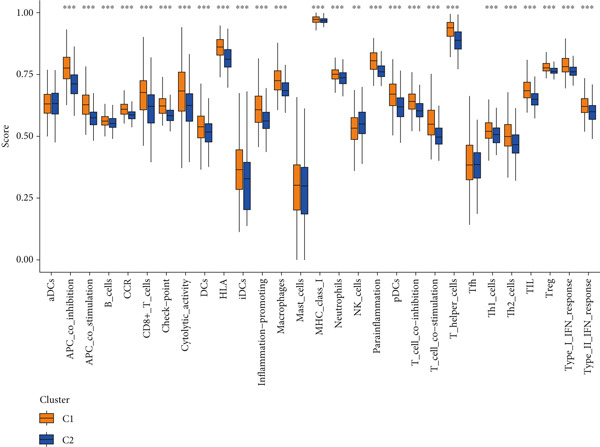
(g)

**Figure:**
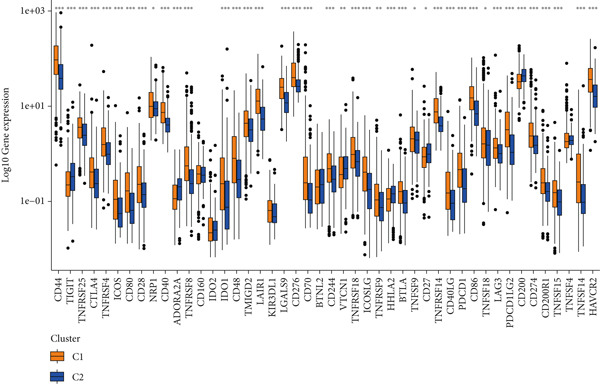
(h)

Cluster 1 was predominantly composed of high‐risk patients, while Cluster 2 mainly included low‐risk individuals (Figure 8c). K‐M analysis demonstrated significantly prolonged OS in Cluster 2 compared with Cluster 1 (Figure 8e). PCA and t‐SNE dimensionality reduction analyses confirmed clear separation between clusters (Supporting Information 10: Figure S9b–e). TME analysis revealed that stromal, immune, and ESTIMATE scores were all higher in Cluster 1 than in Cluster 2 (Figure 8f) along with significant differences in immune cell infiltration, immune‐related pathways, and functions between the clusters (Supporting Information 10: Figure S9a,f). Immune checkpoint analysis indicated that multiple immune checkpoint genes were more highly expressed in Cluster 1 (Figure 8h).

### 3.8. Identifying LINC02542 as a Prognostic Biomarker for Glioma

We used the GEPIA2 online platform to analyze expression differences between glioma and normal tissues. Significant differences were noted in LINC02542 expression in both LGG and GBM (Figure 9a). K‐M survival analysis indicated that patients with glioma with high LINC02542 expression had significantly shorter survival than those with low expression (Figure 9b). Subcellular localization analysis using lncLocator revealed that LINC02542 is primarily located in the cytoplasm (Supporting Information 1: Table S4). Subsequently, we used four siRNAs to inhibit LINC02542 in U251 and U87 cell lines; however, single LINC02542 siRNA transfections were inefficient. Paired siRNA combinations effectively silenced LINC02542 expression (Figure 9c). CCK‐8 and colony formation assays demonstrated that LINC02542 knockdown significantly suppressed glioma cell proliferation (Figure 9d,e). Furthermore, Transwell assays confirmed that LINC02542 inhibition reduced the migration and invasion capabilities of U251 and U87 cells (Figure 9f,g). Overall, these findings reveal that LINC02542 promotes glioma cell growth, migration, and invasion in vitro, indicating its potential as a therapeutic target in glioma.

Figure 9Knockdown of LINC02542 suppresses viability, migration, and invasion in U251 and U87 glioma cells. (a) LINC02542 expression in glioma versus normal brain tissues. (b) Kaplan–Meier overall survival stratified by LINC02542 expression. (c) RT–qPCR validation of LINC02542 knockdown. (d, e) CCK‐8 cell viability in si‐NC and si‐LINC02542 groups. (f, g) Transwell assays of migration and invasion in si‐NC and si‐LINC02542 groups. Data are mean ± SD (*n* = 3 independent experiments).  ^∗∗∗^ *p* < 0.001. si‐NC, negative‐control siRNA.

**Figure:**
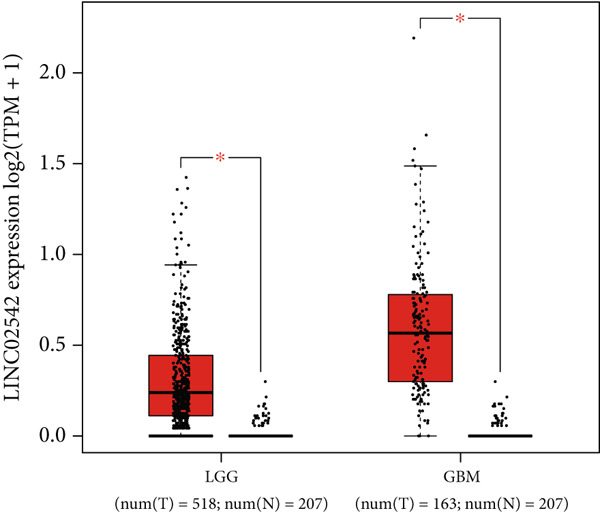
(a)

**Figure:**
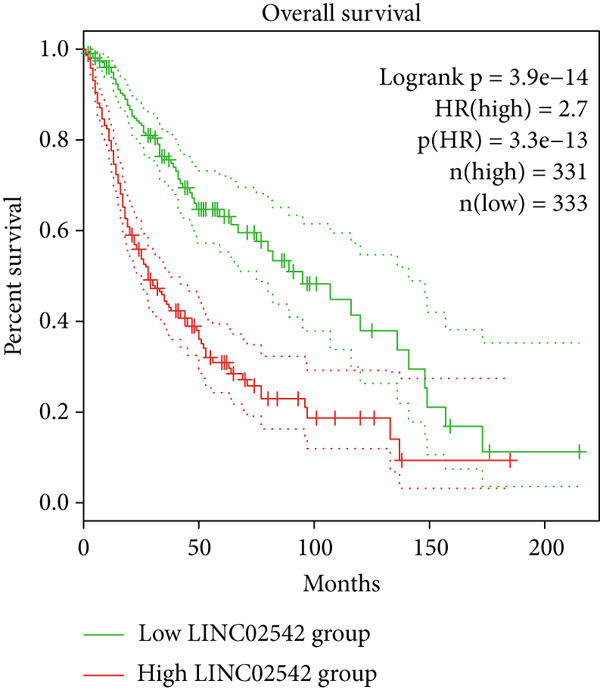
(b)

**Figure:**
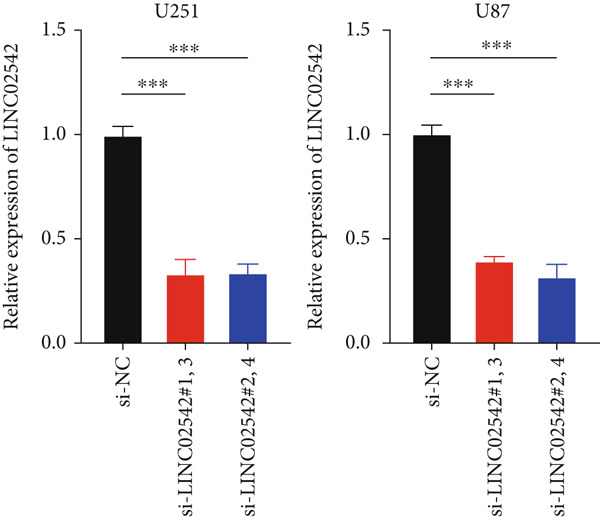
(c)

**Figure:**
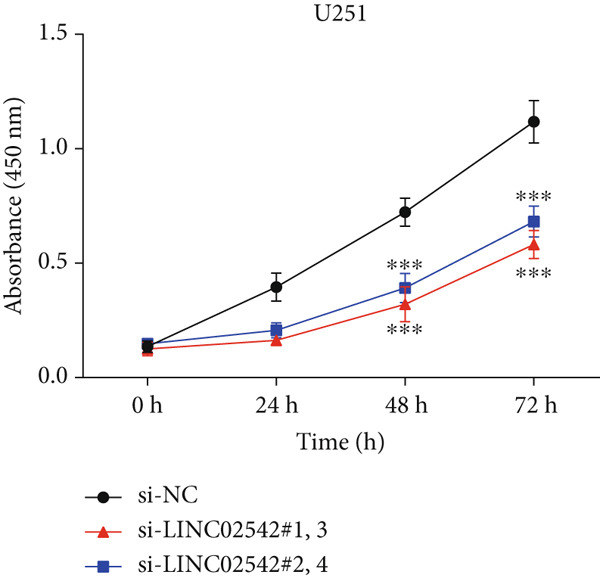
(d)

**Figure:**
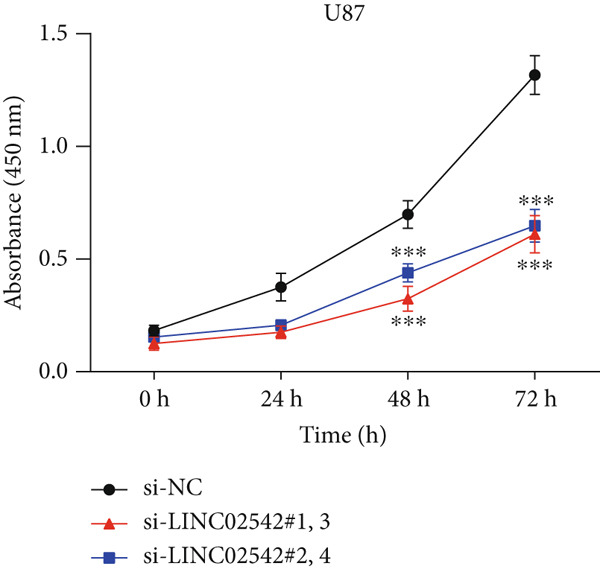
(e)

**Figure:**
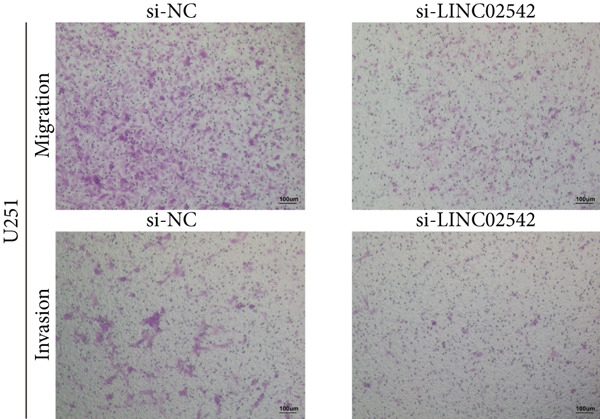
(f)

**Figure:**
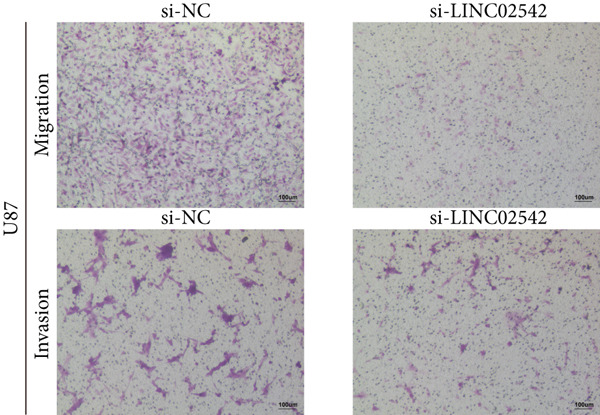
(g)

## 4. Discussion

This study demonstrates the prognostic value of DRLs in gliomas. Using the TCGA dataset, we identified seven DRLs associated with prognosis and developed a DRL‐based risk model. In both the training and validation sets, the model exhibited strong predictive performance for OS and PFS in patients with glioma. Stratified analyses based on clinical characteristics confirmed that low‐risk patients had significantly better survival outcomes, regardless of gender, age, and tumor grade. Additionally, we investigated differences in immune‐related characteristics and TMB between the two risk groups, as well as potential drugs for chemotherapy and targeted therapy based on DRL signatures. Consensus clustering based on DRL expression levels defined molecular subtypes with distinct immune profiles. Lastly, we identified the biological role of LINC02542 in gliomas, where its knockdown significantly inhibited the proliferation, migration, and invasion of glioma cells.

Gliomas are immunologically “cold” tumors, contributing to the limited efficacy of immunotherapy [32]. In this study, DEGs between risk groups were strongly associated with immune‐related biological processes, such as antigen processing and immune receptor activity. Immune cell infiltration analysis revealed differential infiltration of various immune cells between the high‐ and low‐risk groups as well as elevated macrophage infiltration in the high‐risk group. These macrophages, circulating monocyte‐derived macrophages and microglia, are known as tumor‐associated macrophages (TAMs) [33]. A large infiltration of TAMs is associated with poor glioma prognosis [34]. Inhibiting TAMs differentiation into M2 macrophages or depleting TAMs can inhibit glioma progression [35] making TAMs a promising immunotherapeutic agent for gliomas. Notably, high‐risk patients exhibited reduced infiltration of activated NK cells and resting NK cells, indicating NK cell dysfunction is common in the glioma TME, particularly after radiotherapy and chemotherapy [36], resulting from immunosuppressive regulation by TAMs, myeloid‐derived suppressor cells, and Tregs cells [37]. Dysfunctional NK cells not only fail to mediate cytotoxicity but may also promote tumor progression [38].

Immune checkpoint blockade, a cornerstone of modern cancer therapy, has shown limited therapeutic benefits in gliomas [39–41], possibly due to the phenotypic diversity and heterogeneity of gliomas. Our study indicates that high‐risk patients had greater TIDE scores, suggesting stronger tumor immune evasion capabilities. Correspondingly, immune checkpoint analysis showed that high expression of various immune checkpoint genes, such as *IDO1*, *CTLA4*, and *CD274* (PD‐L1), was upregulated in the high‐risk group. CTLA‐4 is a critical immune checkpoint receptor, and its overexpression in tumor and immune cells can lead to tumor progression [42]. CTLA‐4 can inhibit antitumor responses in the glioma TME. CTLA‐4 has been shown to activate microglia Th1 interactions, enhancing microglial phagocytosis and antitumor activity [43]. Furthermore, triple blockade of *IDO*, *CTLA-4*, and *PD-L1* can effectively deplete regulatory T cells (Tregs) and prolong long‐term survival in glioma animal models [44]. However, CTLA‐4‐targeting therapies are limited by CTLA‐4’s inability to cross the blood–brain barrier (BBB) [45]. Additionally, *PD-L1* expression was significantly correlated with several DRLs, suggesting that the DRL model may inform the selection of targeted therapies against immune checkpoints.

Although TMB is a recognized biomarker for immunotherapy response in certain cancers [46], its role in gliomas remains unclear. High TMB has not been associated with better survival outcomes in response to immunotherapy, which may be due to the overall lack of efficacy of immunotherapy in gliomas [47]. Our study showed that the high‐risk group has a higher total TMB count, along with greater mutations in *PTEN*, *EGFR*, and *TTN*. In contrast, *IDH1* and *ATRX* mutations were more prevalent in the low‐risk group. PTEN mutations are associated with high invasiveness and poor prognosis in gliomas. Recent studies have focused more on the regulation of the immune microenvironment by PTEN and its impact on immunotherapy. *PTEN* deficiency in GBM enhances macrophage infiltration through the YAP1‐LOX‐*β*1 integrin‐PYK2 axis, and macrophages secrete SPP1 to promote GBM survival [48]. A study involving 66 adult patients with GBM treated with PD‐1 inhibitors found a significant enrichment of *PTEN* mutations in tumors that did not respond to treatment [49]. *EGFR* amplification is very common in GBM. Abnormal EGFR signaling can lead to tumor behaviors such as growth and invasion [50]. EGFR‐targeted therapies have yielded limited benefits due to resistance mechanisms, including target independence and target compensation; target independence involves glioma cells regulating EGFR expression through extrachromosomal DNA, and target compensation involves activating compensatory pathways independent of EGFR signaling to resist inhibition [51]. Targeting both resistance mechanisms with a combination of EGFR inhibitors and other downstream blockers for multitargeted therapy may yield better results [52]. ATRX is an important prognostic factor in gliomas, commonly seen in IDH‐mutant astrocytomas [53]. In gliomas with *IDH1* R132H and no 1p/19q codeletion, ATRX loss is associated with improved OS and PFS [54]. In combination with *IDH1* mutations, *ATRX* loss contributes to the alternative lengthening of telomeres phenotype in gliomas, facilitating DNA repair and resistance to ionizing radiation [55]. Additionally, *ATRX* loss induces BRD‐mediated immunosuppressive and immune escape mechanisms in *IDH1* R132H or P53‐mutant astrocytomas [56].

Chemotherapy response prediction is critical for glioma management. Current first‐line regimens include the Stupp protocol (temozolomide) and the PCV regimen (procarbazine, lomustine, and vincristine) [3]. In our study, high‐risk patients showed increased sensitivity to 5‐fluorouracil, cisplatin, and gemcitabine. However, BBB penetration remains a major limitation for 5‐fluorouracil and cisplatin. Novel delivery systems, such as gold nanocages, lipophilic conjugates, and stereotactic microsphere implants, have shown potential in preclinical studies [57, 58]. Gemcitabine, an MGMT‐independent cytotoxic agent and radiosensitizer with immunomodulatory effects, has shown a favorable safety profile in glioma [59], though its efficacy remains inconclusive in clinical trials. Our findings suggest that high‐risk patients may benefit from gemcitabine‐based therapies. Additionally, these patients exhibited higher sensitivity to targeted inhibitors of MEK/ERK, PI3K/AKT/mTOR, BET, DNA repair pathways, and HGFR, supporting the utility of the DRL model in guiding personalized treatment.

Consistent with our findings, prior studies have developed DRL‐based prognostic models in gliomas. Guo et al. and Chen et al. constructed models with 8 and 6 DRLs, respectively, both based on 10 DRGs [60, 61]. In contrast, our model incorporated a broader and more comprehensive set of DRGs, resulting in superior predictive performance, as evidenced by 1‐, 3‐, and 5‐year AUC values of 0.867, 0.914, and 0.888, respectively. Moreover, our study is the first to elucidate the functional role of LINC02542. LINC02542 was significantly upregulated in gliomas and negatively correlated with OS. Functional assays revealed that silencing LINC02542 markedly suppressed glioma cell proliferation, migration, and invasion, suggesting its potential as a prognostic biomarker and therapeutic target.

This study has several limitations. The immune landscape and drug sensitivity analyses are based on bioinformatic predictions and require experimental validation. Additionally, the precise molecular mechanisms by which LINC02542 contributes to glioma progression as well as its association with disulfidptosis remain to be elucidated. These questions will be addressed in future studies.

## 5. Conclusion

In this study, we established a prognostic model based on seven DRLs, which can predict the prognosis of patients with glioma and potentially guide immune and targeted therapies. Functional experiments showed that inhibition of LINC02542 suppressed glioma cell growth, migration, and invasion. These findings highlight LINC02542 as a promising biomarker and potential therapeutic target in glioma.

## Ethics Statement

This study involved the analysis of data from publicly available databases and previously published studies, all of which had obtained appropriate ethical approvals. Experimental procedures involving human glioma cell lines (U251 and U87) were conducted in accordance with the institutional biosafety and ethical guidelines. No experiments involving human participants or animals were performed by the authors.

## Disclosure

A preprint version of this manuscript was previously posted on Research Square (10.21203/rs.3.rs-5340635/v1). All authors have read and approved the final version of the manuscript.

## Conflicts of Interest

The authors declare no conflicts of interest.

## Author Contributions

Bin Dong and Yanqin Yang contributed to the conceptualization and supervision of the study. Taiyao Li and Ying Cao carried out the experiments and data analysis. Jie Wang and Xiaoyuan Tian conducted the cell experiments and contributed to the original draft. Bin Dong and Taiyao Li were responsible for writing and revising the manuscript. Taiyao Li, Ying Cao, and Jie Wang have contributed equally to this work and share first authorship.

## Funding

The study is supported by the National Natural Science Foundation of China, 10.13039/501100001809 (81672968).

## Supporting Information

Additional supporting information can be found online in the Supporting Information section.

## Supporting information


**Supporting Information 1** Table S1: Twenty‐four disulfidptosis‐related genes. Table S2: siRNA sequences. Table S3: Primer list for PCR. Table S4: lncLocator predicts subcellular localization of LINC02542.

**Supporting Information 2** Figure S1: Identification and establishment of a prognostic signature for DRLs. (a) Sankey diagram showing the expression correlation between 24 DRGs and 621 DRLs. (b) LASSO regression identified 18 prognostic DRLs. (c) Mean squared error determined by 10‐fold cross‐validation as a function of log(*λ*). (d) Regression coefficients of the 18 DRLs selected by LASSO as a function of log(*λ*), with each line representing a DRL. (e) Correlation analysis between the seven DRLs used for model construction and DRGs.  ^∗^ *p* < 0.05,  ^∗∗^ *p* < 0.01, and  ^∗∗∗^ *p* < 0.001.

**Supporting Information 3** Figure S2: Kaplan–Meier analysis of PFS. (a) Training set. (b) Test set. (c) Full dataset. (d) Correlation between the prognostic DRL model and WHO Grade 4 glioma.

**Supporting Information 4** Figure S3: Subgroup stratification according to patient clinical characteristics. (a) Distribution of patients with different clinical characteristics. (b–d) Summary of age, gender, and tumor grade distribution.

**Supporting Information 5** Figure S4: PCA validation of the spatial grouping performance of the DRL model. (a) All genes. (b) DRGs. (c) DRLs. (d) DRL model.

**Supporting Information 6** Figure S5: Functional enrichment analysis. (a) GO enrichment showing that DEGs are enriched in biological processes (BP), cellular components (CC), and molecular functions (MF). (b, c) GSEA results showing KEGG pathway enrichment. (d, e) GSEA results showing GO term enrichment. (f, g) GSVA analysis showing correlations between model DRLs and multiple cancer‐related pathways from KEGG and Hallmark datasets.  ^∗^ *p* < 0.05,  ^∗∗^ *p* < 0.01, and  ^∗∗∗^ *p* < 0.001.

**Supporting Information 7** Figure S6: Immune‐related analysis between high‐ and low‐risk groups. (a) Heatmap showing differential immune cell infiltration based on seven algorithms. (b) Correlation analysis between seven DRLs and immune checkpoint genes. (c) GSVA showing differences in immune function pathways between the two risk groups.  ^∗^ *p* < 0.05,  ^∗∗^ *p* < 0.01, and  ^∗∗∗^ *p* < 0.001.

**Supporting Information 8** Figure S7: Differential analysis of PD‐L1 expression in patients with high and low expression of DRLs ( ^∗^ *p* < 0.05,  ^∗∗∗^ *p* < 0.001).

**Supporting Information 9** Figure S8: Drug sensitivity analysis based on IC_50_ values between high‐ and low‐risk groups. (a) Standard chemotherapy drugs. (b) MEK/ERK pathway inhibitors. (c) PI3K/AKT/mTOR pathway inhibitors. (d) Protein kinase inhibitors. (e) BET inhibitors. (f) DNA repair inhibitors. (g) HGFR inhibitors.

**Supporting Information 10** Figure S9: Differential analysis between two clusters identified by consensus clustering. (a) Heatmap showing immune cell infiltration differences between clusters. (b, c) PCA dimensionality reduction of clusters and risk groups showing their differences. (d, e) t‐SNE dimensionality reduction of clusters and risk groups showing their differences. (f) GSVA showing differences in immune function pathways between clusters.

## Data Availability

The data that support the findings of this study are available in The Cancer Genome Atlas at https://www.tcga.org/, reference number TCGA‐LGG, TCGA‐GBM. These data were derived from the following resources available in the public domain: TCGA‐LGG, https://portal.gdc.cancer.gov/projects/TCGA-LGG; TCGA‐GBM, https://portal.gdc.cancer.gov/projects/TCGA-GBM.
