# Supplementary material for: A Novel Signature of Disulfidptosis‐Related lncRNAs Predicts Prognosis in Glioma: Evidence From Bioinformatic Analysis and Experiments
Source: Int J Genomics. 2025 Oct 13;2025:5573323. doi: 10.1155/ijog/5573323 (PMC12517204; doi:10.1155/ijog/5573323)
Supplement: Supplementary file 1 — Supporting Information 1 Table S1: Twenty‐four disulfidptosis‐related genes. Table S2: siRNA sequences. Table S3: Primer list for PCR. Table S4: lncLocator predicts subcellular localization of LINC02542. [file IJOG-2025-5573323-s002.docx]

Supplementary Table S1: 24 disulfidptosis-related genes

| ACTN4 | ACTB | CD2AP | CAPZB | DSTN |
| --- | --- | --- | --- | --- |
| FLNA | FLNB | INF2 | IQGAP1 | MYH10 |
| MYL6 | MYH9 | PDLIM1 | TLN1 | SLC7A11 |
| SLC3A2 | RPN1 | NCKAP1 | NUBPL | NDUFA11 |
| LRPPRC | OXSM | GYS1 | NDUFS1 |  |

Supplementary Table S2: siRNA sequences

| Gene Name | Sence | Antisence |
| --- | --- | --- |
| si-NC | UUCUCCGAACGUGUCACGUTT | ACGUGACACGUUCGGAGAATT |
| si-LINC02542#1 | GCUGUCACCCAUGCCUCUUTT | AAGAGGCAUGGGUGACAGCTT |
| si-LINC02542#2 | GCCAGGAUCCUGGUUGGUATT | UACCAACCAGGAUCCUGGCTT |
| si-LINC02542#3 | GCCCUUAGCAGAGAACCAUTT | AUGGUUCUCUGCUAAGGGCTT |
| si-LINC02542#4 | GCCUGCAAAUUGUAAUGAATT | UUCAUUACAAUUUGCAGGCTT |

Supplementary Table S3: Primer list for PCR

| Gene Name | Forward | Reverse |
| --- | --- | --- |
| LINC02542 | CCCTTTGCTATTCTGCTCCAGTGTTTG | TCCACCAGCCCTACCTTTCCTTTCC |
| HMBS | GGAATGTTACGAGCAGTGATGC | CCTGACTGGAGGAGTCTGGAGT |

Supplementary Table S4 LncLocator predicts subcellular localization of LINC02542

| Subcellular locations | Score |
| --- | --- |
| Cytoplasm | 0.878715906 |
| Nucleus | 0.063929563 |
| Ribosome | 0.020571293 |
| Cytosol | 0.033696765 |
| Exosome | 0.003086472 |
