# Supplementary figures and images for: A Novel Signature of Disulfidptosis‐Related lncRNAs Predicts Prognosis in Glioma: Evidence From Bioinformatic Analysis and Experiments
Source: Int J Genomics. 2025 Oct 13;2025:5573323. doi: 10.1155/ijog/5573323 (PMC12517204; doi:10.1155/ijog/5573323)

(a)

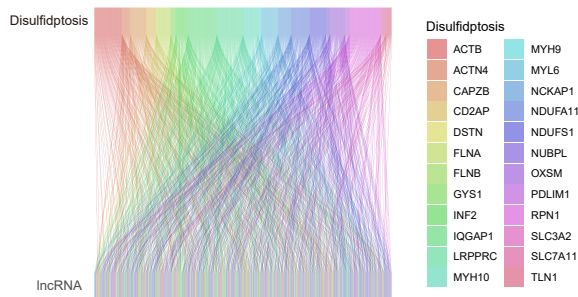

(b)

|            | pvalue | Hazard ratio       |
|------------|--------|--------------------|
| AC010884.1 | <0.001 | 0.127(0.080-0.202) |
| DNAJC3-DT  | <0.001 | 0.396(0.297-0.529) |
| AL592295.6 | <0.001 | 0.324(0.265-0.396) |
| AC010168.2 | <0.001 | 5.730(3.787-8.672) |
| AC002456.1 | <0.001 | 3.280(2.559-4.205) |
| LNCOG      | <0.001 | 2.492(2.016-3.080) |
| LINC02542  | <0.001 | 3.694(2.526-5.403) |
| AC073593.2 | <0.001 | 4.984(3.629-6.846) |
| FAM53B-AS1 | <0.001 | 1.968(1.340-2.889) |
| AL390755.1 | <0.001 | 1.713(1.547-1.897) |
| AC120036.4 | <0.001 | 0.329(0.268-0.404) |
| AC026401.3 | <0.001 | 2.097(1.802-2.441) |
| BX322234.1 | <0.001 | 4.451(3.263-6.072) |
| LEF1-AS1   | <0.001 | 5.414(3.995-7.336) |
| AC010273.3 | <0.001 | 2.404(2.018-2.864) |
| CRNDE      | <0.001 | 2.107(1.816-2.445) |
| AC008875.3 | <0.001 | 1.986(1.700-2.320) |
| AL138479.2 | <0.001 | 0.179(0.116-0.277) |

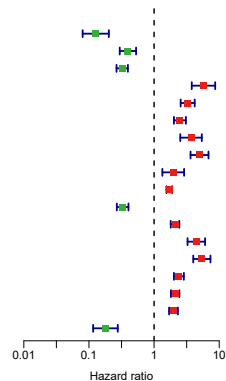

(c)

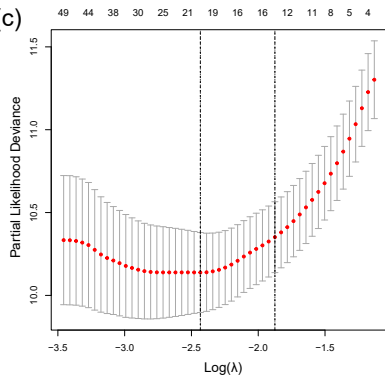

(d)

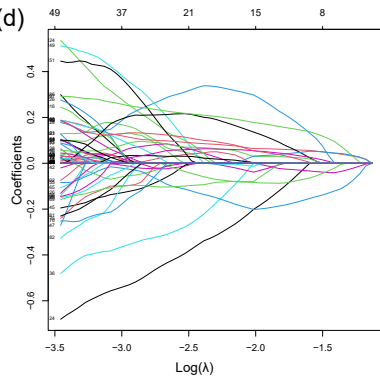

(e)

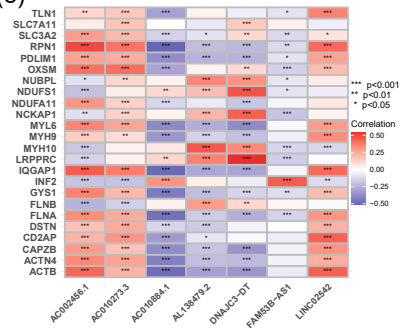

Supplement: Supplementary file 2 — Supporting Information 2 Figure S1: Identification and establishment of a prognostic signature for DRLs. (a) Sankey diagram showing the expression correlation between 24 DRGs and 621 DRLs. (b) LASSO regression identified 18 prognostic DRLs. (c) Mean squared error determined by 10‐fold cross‐validation as a function of log(λ). (d) Regression coefficients of the 18 DRLs selected by LASSO as a function of log(λ), with each line representing a DRL. (e) Correlation analysis between the seven DRLs used for model construction and DRGs. ∗ p < 0.05, ∗∗ p < 0.01, and ∗∗∗ p < 0.001. [file IJOG-2025-5573323-s009.pdf]

(a)

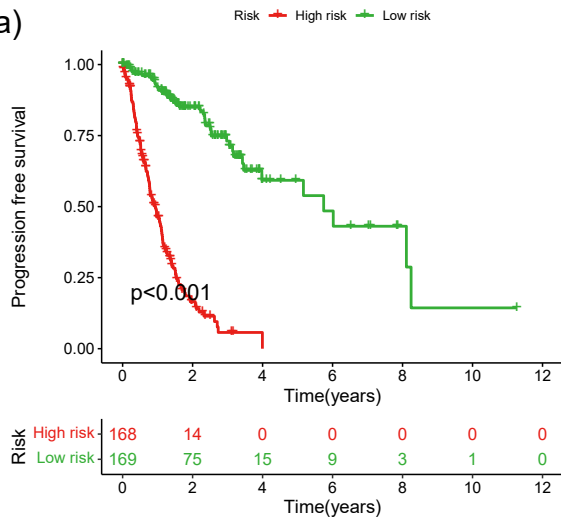

(b)

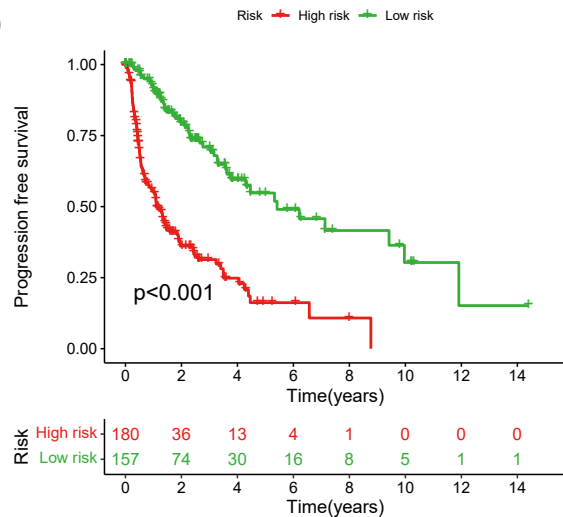

(c)

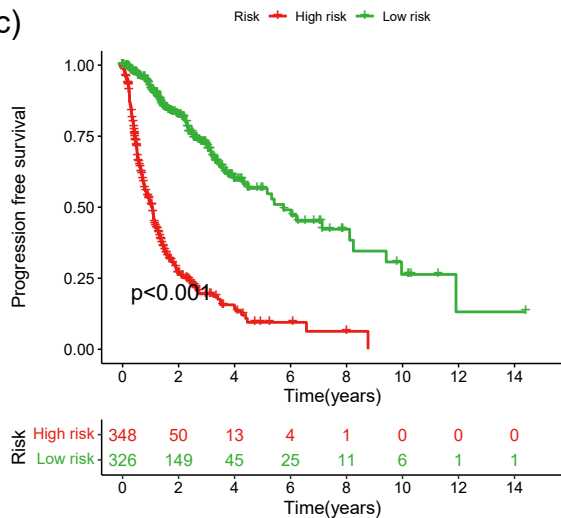

(d)

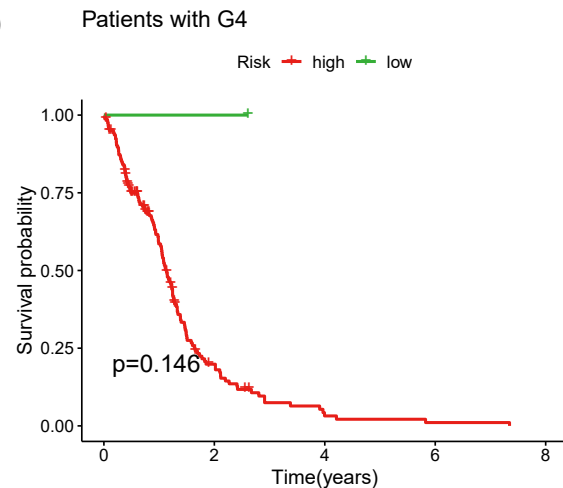

Supplement: Supplementary file 3 — Supporting Information 3 Figure S2: Kaplan–Meier analysis of PFS. (a) Training set. (b) Test set. (c) Full dataset. (d) Correlation between the prognostic DRL model and WHO Grade 4 glioma. [file IJOG-2025-5573323-s003.pdf]

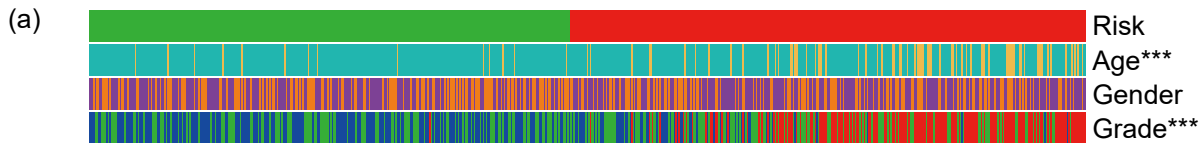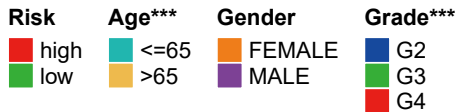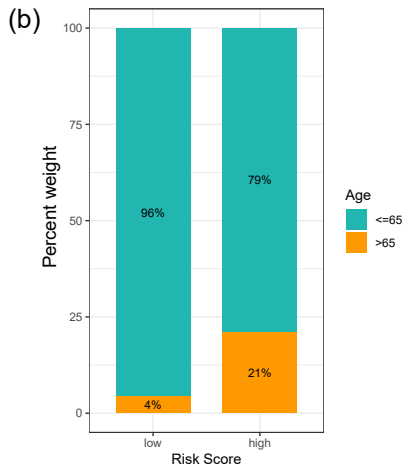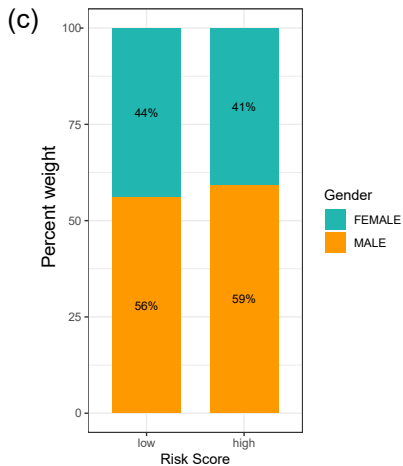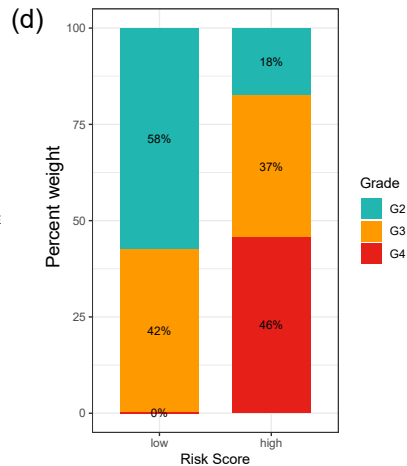

Supplement: Supplementary file 4 — Supporting Information 4 Figure S3: Subgroup stratification according to patient clinical characteristics. (a) Distribution of patients with different clinical characteristics. (b–d) Summary of age, gender, and tumor grade distribution. [file IJOG-2025-5573323-s007.pdf]

(a)

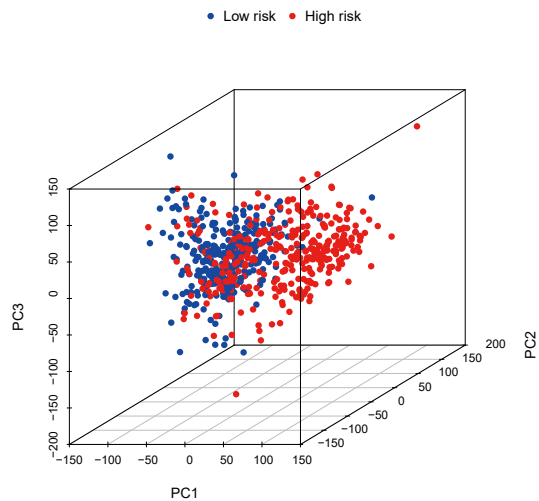

(b)

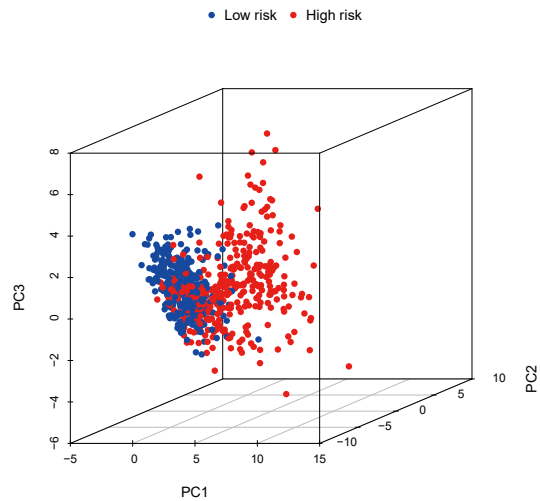

(c)

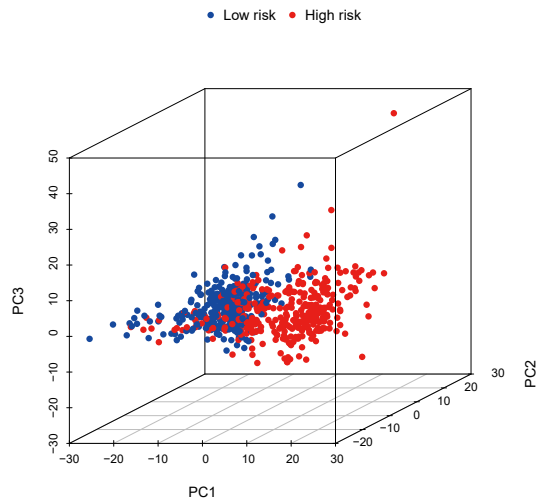

(d)

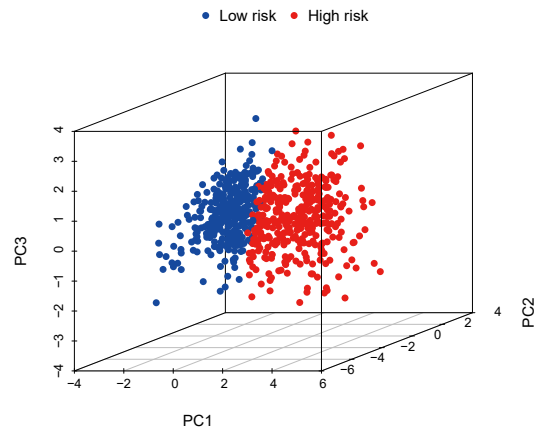

Supplement: Supplementary file 5 — Supporting Information 5 Figure S4: PCA validation of the spatial grouping performance of the DRL model. (a) All genes. (b) DRGs. (c) DRLs. (d) DRL model. [file IJOG-2025-5573323-s006.pdf]

(a)

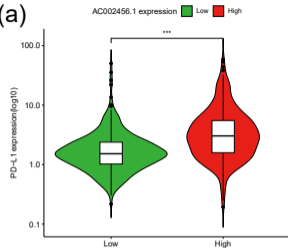

(b)

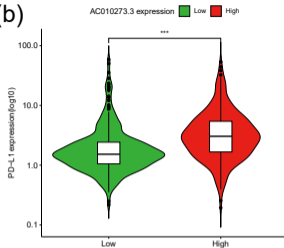

(c)

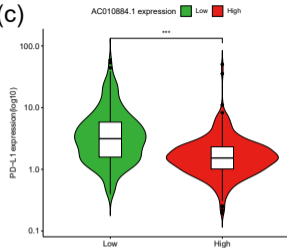

(d)

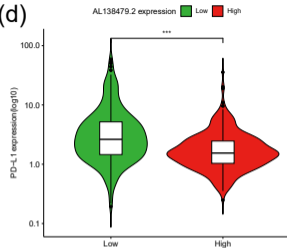

(e)

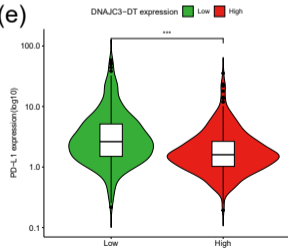

(f)

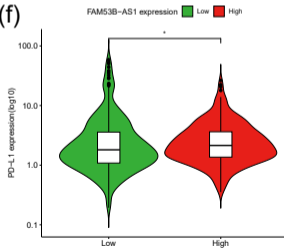

(g)

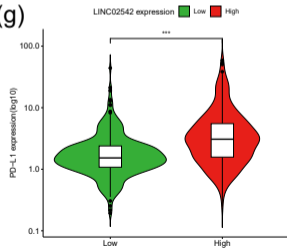

(h)

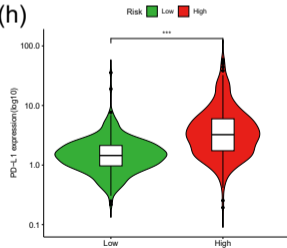

Supplement: Supplementary file 8 — Supporting Information 8 Figure S7: Differential analysis of PD‐L1 expression in patients with high and low expression of DRLs ( ∗ p < 0.05, ∗∗∗ p < 0.001). [file IJOG-2025-5573323-s001.pdf]

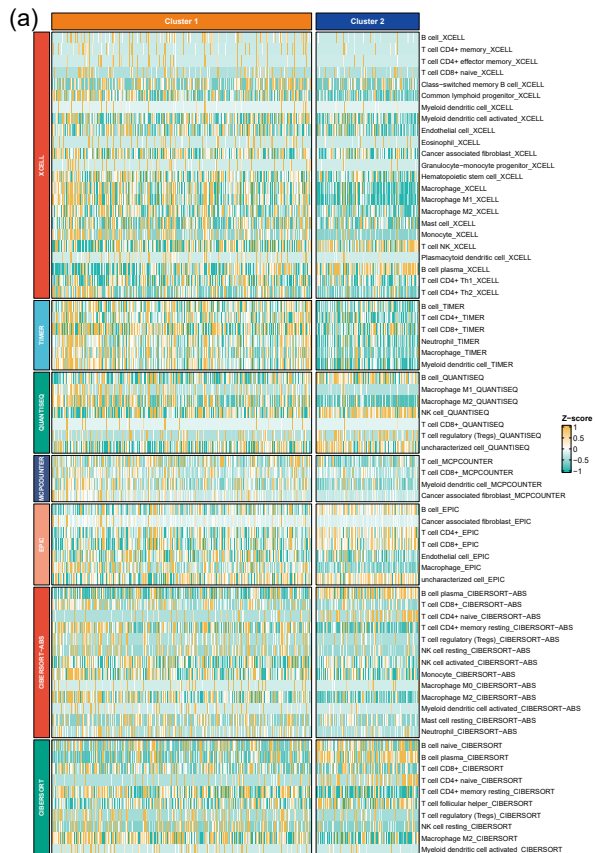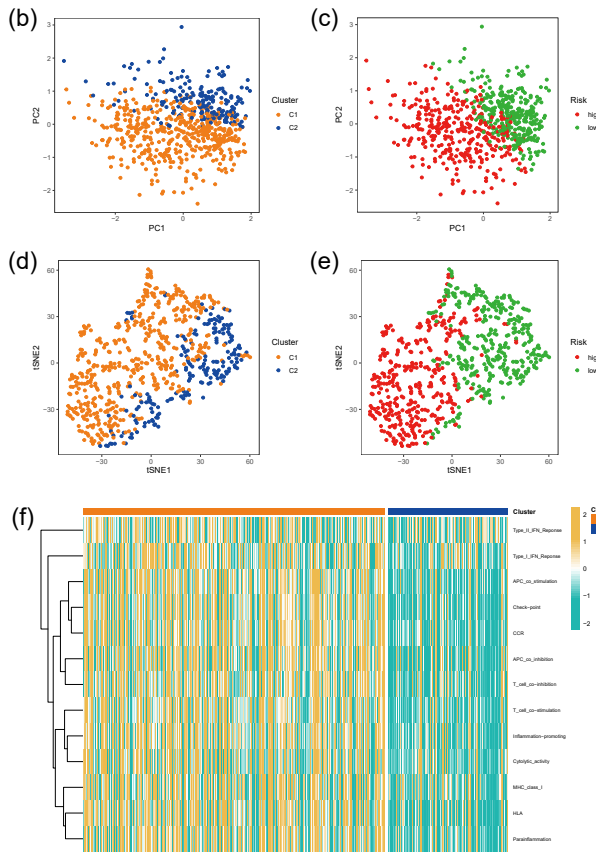

Supplement: Supplementary file 10 — Supporting Information 10 Figure S9: Differential analysis between two clusters identified by consensus clustering. (a) Heatmap showing immune cell infiltration differences between clusters. (b, c) PCA dimensionality reduction of clusters and risk groups showing their differences. (d, e) t‐SNE dimensionality reduction of clusters and risk groups showing their differences. (f) GSVA showing differences in immune function pathways between clusters. [file IJOG-2025-5573323-s010.pdf]
