# Supplementary material for: A Novel Signature of Disulfidptosis‐Related lncRNAs Predicts Prognosis in Glioma: Evidence From Bioinformatic Analysis and Experiments
Source: Int J Genomics. 2025 Oct 13;2025:5573323. doi: 10.1155/ijog/5573323 (PMC12517204; doi:10.1155/ijog/5573323)
Supplement: Supplementary file 9 — Supporting Information 9 Figure S8: Drug sensitivity analysis based on IC50 values between high‐ and low‐risk groups. (a) Standard chemotherapy drugs. (b) MEK/ERK pathway inhibitors. (c) PI3K/AKT/mTOR pathway inhibitors. (d) Protein kinase inhibitors. (e) BET inhibitors. (f) DNA repair inhibitors. (g) HGFR inhibitors. [file IJOG-2025-5573323-s005.pdf]

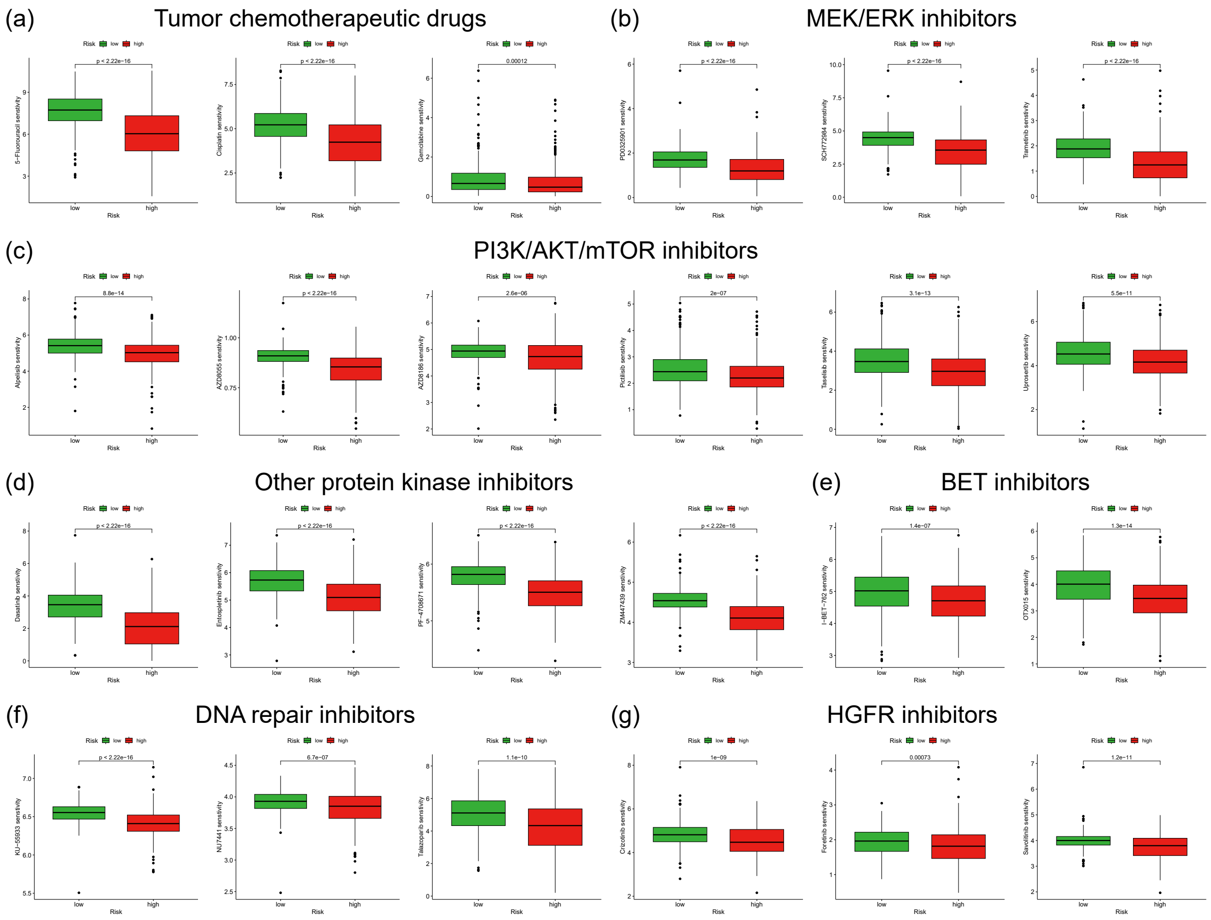
(a) Tumor chemotherapeutic drugs
(b) MEK/ERK inhibitors
(c) PI3K/AKT/mTOR inhibitors
(d) Other protein kinase inhibitors
(e) BET inhibitors
(f) DNA repair inhibitors
(g) HGFR inhibitors
Risk low high
5-Fluorouracil sensitivity p < 2.22e-16
Cisplatin sensitivity p < 2.22e-16
Gemcitabine sensitivity 0.00012
PD0325901 sensitivity p < 2.22e-16
SCH772984 sensitivity p < 2.22e-16
Trametinib sensitivity p < 2.22e-16
Alpelisib sensitivity 8.8e-14
AZD8055 sensitivity p < 2.22e-16
AZD8186 sensitivity 2.6e-06
Pictilisib sensitivity 2e-07
Taselisib sensitivity 3.1e-13
Uprosertib sensitivity 5.5e-11
Dasatinib sensitivity p < 2.22e-16
Entospletinib sensitivity p < 2.22e-16
PF-4708671 sensitivity p < 2.22e-16
ZM447439 sensitivity p < 2.22e-16
I-BET-762 sensitivity 1.4e-07
OTX015 sensitivity 1.3e-14
KU-55933 sensitivity p < 2.22e-16
NU7441 sensitivity 6.7e-07
Talazoparib sensitivity 1.1e-10
Crizotinib sensitivity 1e-09
Foretinib sensitivity 0.00073
Savolitinib sensitivity 1.2e-11
