# Supplementary material for: A Novel Signature of Disulfidptosis‐Related lncRNAs Predicts Prognosis in Glioma: Evidence From Bioinformatic Analysis and Experiments
Source: Int J Genomics. 2025 Oct 13;2025:5573323. doi: 10.1155/ijog/5573323 (PMC12517204; doi:10.1155/ijog/5573323)
Supplement: Supplementary file 6 — Supporting Information 6 Figure S5: Functional enrichment analysis. (a) GO enrichment showing that DEGs are enriched in biological processes (BP), cellular components (CC), and molecular functions (MF). (b, c) GSEA results showing KEGG pathway enrichment. (d, e) GSEA results showing GO term enrichment. (f, g) GSVA analysis showing correlations between model DRLs and multiple cancer‐related pathways from KEGG and Hallmark datasets. ∗ p < 0.05, ∗∗ p < 0.01, and ∗∗∗ p < 0.001. [file IJOG-2025-5573323-s008.pdf]

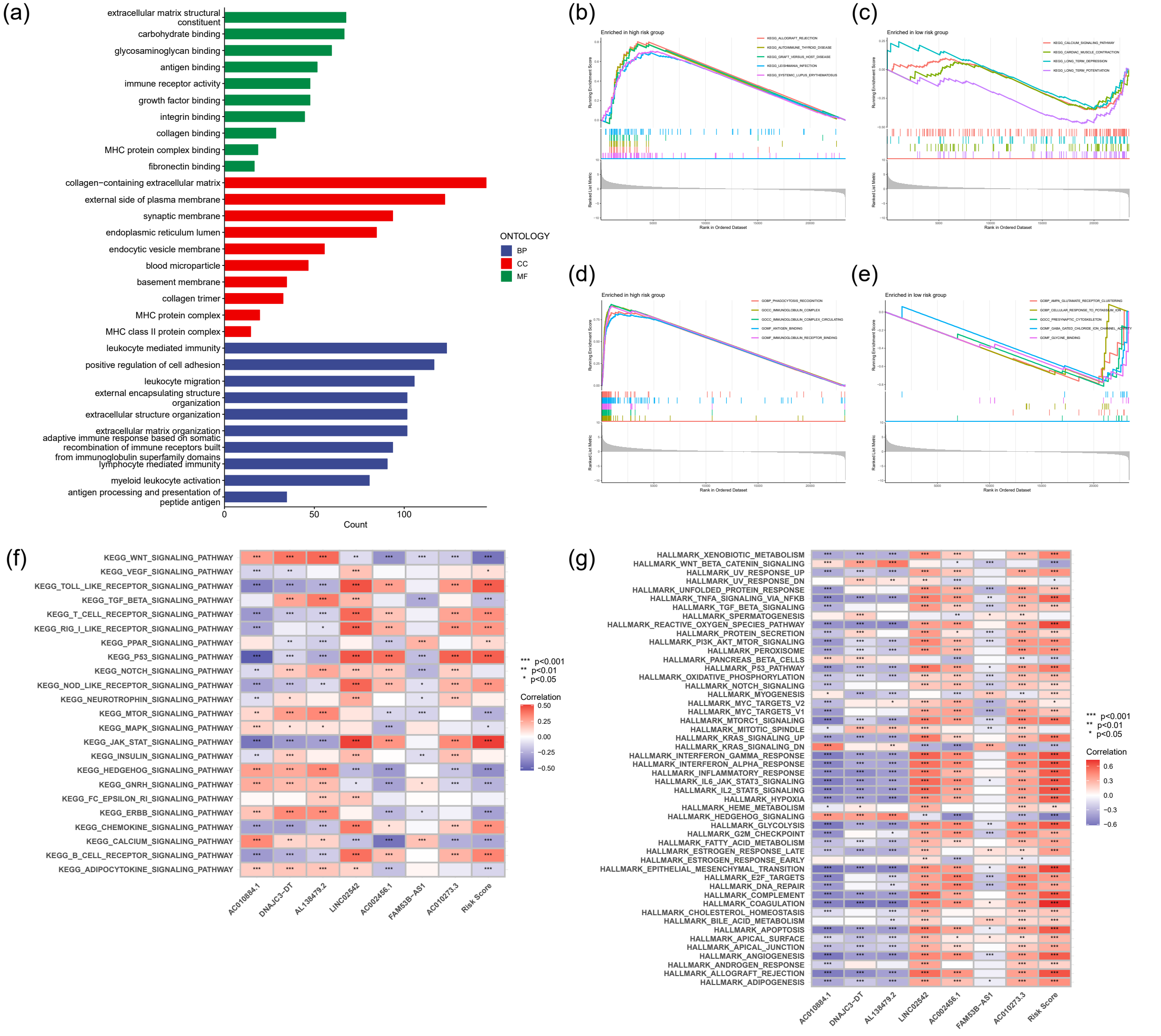

(a)
extracellular matrix structural constituent
carbohydrate binding
glycosaminoglycan binding
antigen binding
immune receptor activity
growth factor binding
integrin binding
collagen binding
MHC protein complex binding
fibronectin binding
collagen−containing extracellular matrix
external side of plasma membrane
synaptic membrane
endoplasmic reticulum lumen
endocytic vesicle membrane
blood microparticle
basement membrane
collagen trimer
MHC protein complex
MHC class II protein complex
leukocyte mediated immunity
positive regulation of cell adhesion
leukocyte migration
external encapsulating structure organization
extracellular structure organization
extracellular matrix organization
adaptive immune response based on somatic recombination of immune receptors built from immunoglobulin superfamily domains
lymphocyte mediated immunity
myeloid leukocyte activation
antigen processing and presentation of peptide antigen
0
50
100
Count
ONTOLOGY
BP
CC
MF
(b)
Enriched in high risk group
KEGG_ALLOGRAFT_REJECTION
KEGG_AUTOIMMUNE_THYROID_DISEASE
KEGG_GRAFT_VERSUS_HOST_DISEASE
KEGG_LEISHMANIA_INFECTION
KEGG_SYSTEMIC_LUPUS_ERYTHEMATOSUS
Running Enrichment Score
Ranked List Metric
Rank in Ordered Dataset
(c)
Enriched in low risk group
KEGG_CALCIUM_SIGNALING_PATHWAY
KEGG_CARDIAC_MUSCLE_CONTRACTION
KEGG_LONG_TERM_DEPRESSION
KEGG_LONG_TERM_POTENTIATION
Running Enrichment Score
Ranked List Metric
Rank in Ordered Dataset
(d)
Enriched in high risk group
GOBP_PHAGOCYTOSIS_RECOGNITION
GOCC_IMMUNOGLOBULIN_COMPLEX
GOCC_IMMUNOGLOBULIN_COMPLEX_CIRCULATING
GOMF_ANTIGEN_BINDING
GOMF_IMMUNOGLOBULIN_RECEPTOR_BINDING
Running Enrichment Score
Ranked List Metric
Rank in Ordered Dataset
(e)
Enriched in low risk group
GOBP_AMPA_GLUTAMATE_RECEPTOR_CLUSTERING
GOBP_CELLULAR_RESPONSE_TO_POTASSIUM_ION
GOCC_PRESYNAPTIC_CYTOSKELETON
GOMF_GABA_GATED_CHLORIDE_ION_CHANNEL_ACTIVITY
GOMF_GLYCINE_BINDING
Running Enrichment Score
Ranked List Metric
Rank in Ordered Dataset
(f)
KEGG_WNT_SIGNALING_PATHWAY
KEGG_VEGF_SIGNALING_PATHWAY
KEGG_TOLL_LIKE_RECEPTOR_SIGNALING_PATHWAY
KEGG_TGF_BETA_SIGNALING_PATHWAY
KEGG_T_CELL_RECEPTOR_SIGNALING_PATHWAY
KEGG_RIG_I_LIKE_RECEPTOR_SIGNALING_PATHWAY
KEGG_PPAR_SIGNALING_PATHWAY
KEGG_P53_SIGNALING_PATHWAY
KEGG_NOTCH_SIGNALING_PATHWAY
KEGG_NOD_LIKE_RECEPTOR_SIGNALING_PATHWAY
KEGG_NEUROTROPHIN_SIGNALING_PATHWAY
KEGG_MTOR_SIGNALING_PATHWAY
KEGG_MAPK_SIGNALING_PATHWAY
KEGG_JAK_STAT_SIGNALING_PATHWAY
KEGG_INSULIN_SIGNALING_PATHWAY
KEGG_HEDGEHOG_SIGNALING_PATHWAY
KEGG_GNRH_SIGNALING_PATHWAY
KEGG_FC_EPSILON_RI_SIGNALING_PATHWAY
KEGG_ERBB_SIGNALING_PATHWAY
KEGG_CHEMOKINE_SIGNALING_PATHWAY
KEGG_CALCIUM_SIGNALING_PATHWAY
KEGG_B_CELL_RECEPTOR_SIGNALING_PATHWAY
KEGG_ADIPOCYTOKINE_SIGNALING_PATHWAY
AC010884.1
DNAJC3−DT
AL138479.2
LINC02542
AC002456.1
FAM53B−AS1
AC010273.3
Risk Score
*** p<0.001
** p<0.01
* p<0.05
Correlation
0.50
0.25
0.00
−0.25
−0.50
(g)
HALLMARK_XENOBIOTIC_METABOLISM
HALLMARK_WNT_BETA_CATENIN_SIGNALING
HALLMARK_UV_RESPONSE_UP
HALLMARK_UV_RESPONSE_DN
HALLMARK_UNFOLDED_PROTEIN_RESPONSE
HALLMARK_TNFA_SIGNALING_VIA_NFKB
HALLMARK_TGF_BETA_SIGNALING
HALLMARK_SPERMATOGENESIS
HALLMARK_REACTIVE_OXYGEN_SPECIES_PATHWAY
HALLMARK_PROTEIN_SECRETION
HALLMARK_PI3K_AKT_MTOR_SIGNALING
HALLMARK_PEROXISOME
HALLMARK_PANCREAS_BETA_CELLS
HALLMARK_P53_PATHWAY
HALLMARK_OXIDATIVE_PHOSPHORYLATION
HALLMARK_NOTCH_SIGNALING
HALLMARK_MYOGENESIS
HALLMARK_MYC_TARGETS_V2
HALLMARK_MYC_TARGETS_V1
HALLMARK_MTORC1_SIGNALING
HALLMARK_MITOTIC_SPINDLE
HALLMARK_KRAS_SIGNALING_UP
HALLMARK_KRAS_SIGNALING_DN
HALLMARK_INTERFERON_GAMMA_RESPONSE
HALLMARK_INTERFERON_ALPHA_RESPONSE
HALLMARK_INFLAMMATORY_RESPONSE
HALLMARK_IL6_JAK_STAT3_SIGNALING
HALLMARK_IL2_STAT5_SIGNALING
HALLMARK_HYPOXIA
HALLMARK_HEME_METABOLISM
HALLMARK_HEDGEHOG_SIGNALING
HALLMARK_GLYCOLYSIS
HALLMARK_G2M_CHECKPOINT
HALLMARK_FATTY_ACID_METABOLISM
HALLMARK_ESTROGEN_RESPONSE_LATE
HALLMARK_ESTROGEN_RESPONSE_EARLY
HALLMARK_EPITHELIAL_MESENCHYMAL_TRANSITION
HALLMARK_E2F_TARGETS
HALLMARK_DNA_REPAIR
HALLMARK_COMPLEMENT
HALLMARK_COAGULATION
HALLMARK_CHOLESTEROL_HOMEOSTASIS
HALLMARK_BILE_ACID_METABOLISM
HALLMARK_APOPTOSIS
HALLMARK_APICAL_SURFACE
HALLMARK_APICAL_JUNCTION
HALLMARK_ANGIOGENESIS
HALLMARK_ANDROGEN_RESPONSE
HALLMARK_ALLOGRAFT_REJECTION
HALLMARK_ADIPOGENESIS
AC010884.1
DNAJC3−DT
AL138479.2
LINC02542
AC002456.1
FAM53B−AS1
AC010273.3
Risk Score
*** p<0.001
** p<0.01
* p<0.05
Correlation
0.6
0.3
0.0
−0.3
−0.6
