# Supplementary material for: A Novel Signature of Disulfidptosis‐Related lncRNAs Predicts Prognosis in Glioma: Evidence From Bioinformatic Analysis and Experiments
Source: Int J Genomics. 2025 Oct 13;2025:5573323. doi: 10.1155/ijog/5573323 (PMC12517204; doi:10.1155/ijog/5573323)
Supplement: Supplementary file 7 — Supporting Information 7 Figure S6: Immune‐related analysis between high‐ and low‐risk groups. (a) Heatmap showing differential immune cell infiltration based on seven algorithms. (b) Correlation analysis between seven DRLs and immune checkpoint genes. (c) GSVA showing differences in immune function pathways between the two risk groups. ∗ p < 0.05, ∗∗ p < 0.01, and ∗∗∗ p < 0.001. [file IJOG-2025-5573323-s004.pdf]

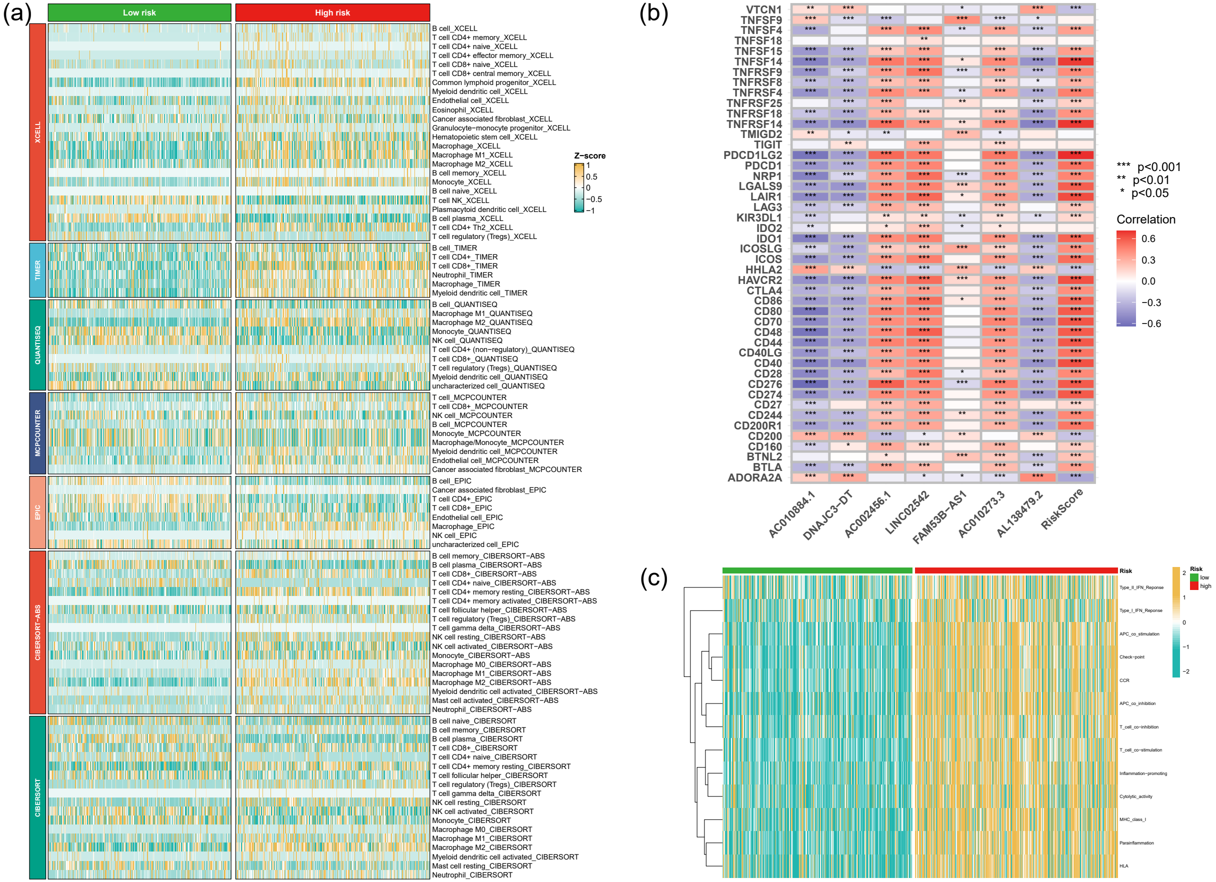

(a)
Low risk
High risk
XCELL
TIMER
QUANTISEQ
MCPCOUNTER
EPIC
CIBERSORT-ABS
CIBERSORT
B cell_XCELL
T cell CD4+ memory_XCELL
T cell CD4+ naive_XCELL
T cell CD4+ effector memory_XCELL
T cell CD8+ naive_XCELL
T cell CD8+ central memory_XCELL
Common lymphoid progenitor_XCELL
Myeloid dendritic cell_XCELL
Endothelial cell_XCELL
Eosinophil_XCELL
Cancer associated fibroblast_XCELL
Granulocyte−monocyte progenitor_XCELL
Hematopoietic stem cell_XCELL
Macrophage_XCELL
Macrophage M1_XCELL
Macrophage M2_XCELL
B cell memory_XCELL
Monocyte_XCELL
B cell naive_XCELL
T cell NK_XCELL
Plasmacytoid dendritic cell_XCELL
B cell plasma_XCELL
T cell CD4+ Th2_XCELL
T cell regulatory (Tregs)_XCELL
B cell_TIMER
T cell CD4+_TIMER
T cell CD8+_TIMER
Neutrophil_TIMER
Macrophage_TIMER
Myeloid dendritic cell_TIMER
B cell_QUANTISEQ
Macrophage M1_QUANTISEQ
Macrophage M2_QUANTISEQ
Monocyte_QUANTISEQ
NK cell_QUANTISEQ
T cell CD4+ (non−regulatory)_QUANTISEQ
T cell CD8+_QUANTISEQ
T cell regulatory (Tregs)_QUANTISEQ
Myeloid dendritic cell_QUANTISEQ
uncharacterized cell_QUANTISEQ
T cell_MCPCOUNTER
T cell CD8+_MCPCOUNTER
NK cell_MCPCOUNTER
B cell_MCPCOUNTER
Monocyte_MCPCOUNTER
Macrophage/Monocyte_MCPCOUNTER
Myeloid dendritic cell_MCPCOUNTER
Endothelial cell_MCPCOUNTER
Cancer associated fibroblast_MCPCOUNTER
B cell_EPIC
Cancer associated fibroblast_EPIC
T cell CD4+_EPIC
T cell CD8+_EPIC
Endothelial cell_EPIC
Macrophage_EPIC
NK cell_EPIC
uncharacterized cell_EPIC
B cell memory_CIBERSORT−ABS
B cell plasma_CIBERSORT−ABS
T cell CD8+_CIBERSORT−ABS
T cell CD4+ naive_CIBERSORT−ABS
T cell CD4+ memory resting_CIBERSORT−ABS
T cell CD4+ memory activated_CIBERSORT−ABS
T cell follicular helper_CIBERSORT−ABS
T cell regulatory (Tregs)_CIBERSORT−ABS
T cell gamma delta_CIBERSORT−ABS
NK cell resting_CIBERSORT−ABS
NK cell activated_CIBERSORT−ABS
Monocyte_CIBERSORT−ABS
Macrophage M0_CIBERSORT−ABS
Macrophage M1_CIBERSORT−ABS
Macrophage M2_CIBERSORT−ABS
Myeloid dendritic cell activated_CIBERSORT−ABS
Mast cell activated_CIBERSORT−ABS
Neutrophil_CIBERSORT−ABS
B cell naive_CIBERSORT
B cell memory_CIBERSORT
B cell plasma_CIBERSORT
T cell CD8+_CIBERSORT
T cell CD4+ naive_CIBERSORT
T cell CD4+ memory resting_CIBERSORT
T cell follicular helper_CIBERSORT
T cell regulatory (Tregs)_CIBERSORT
T cell gamma delta_CIBERSORT
NK cell resting_CIBERSORT
NK cell activated_CIBERSORT
Monocyte_CIBERSORT
Macrophage M0_CIBERSORT
Macrophage M1_CIBERSORT
Macrophage M2_CIBERSORT
Myeloid dendritic cell activated_CIBERSORT
Mast cell resting_CIBERSORT
Neutrophil_CIBERSORT
Z−score
1
0.5
0
−0.5
−1
(b)
VTCN1
TNFSF9
TNFSF4
TNFSF18
TNFSF15
TNFSF14
TNFRSF9
TNFRSF8
TNFRSF4
TNFRSF25
TNFRSF18
TNFRSF14
TMIGD2
TIGIT
PDCD1LG2
PDCD1
NRP1
LGALS9
LAIR1
LAG3
KIR3DL1
IDO2
IDO1
ICOSLG
ICOS
HHLA2
HAVCR2
CTLA4
CD86
CD80
CD70
CD48
CD44
CD40LG
CD40
CD28
CD276
CD274
CD27
CD244
CD200R1
CD200
CD160
BTNL2
BTLA
ADORA2A
AC010884.1
DNAJC3−DT
AC002456.1
LINC02542
FAM53B−AS1
AC010273.3
AL138479.2
RiskScore
*** p<0.001
** p<0.01
* p<0.05
Correlation
0.6
0.3
0.0
−0.3
−0.6
(c)
Risk
Type_II_IFN_Reponse
Type_I_IFN_Reponse
APC_co_stimulation
Check−point
CCR
APC_co_inhibition
T_cell_co−inhibition
T_cell_co−stimulation
Inflammation−promoting
Cytolytic_activity
MHC_class_I
Parainflammation
HLA
2
1
0
−1
−2
Risk
low
high
